# Plasma-reinforced dual-crosslinked Pueraria hydrogel coating for synergistic atherosclerosis intervention

**DOI:** 10.1016/j.mtbio.2025.102313

**Published:** 2025-09-14

**Authors:** Jingyue Wang, Ge Yin, Leila Mamizadeh Janghour, Sheng Dai, Behnam Akhavan, Maolin Sun, Ansha Zhao

**Affiliations:** a Institute of Biomedical Engineering, College of Medicine, Southwest Jiaotong University, Chengdu, 610031, Sichuan, China; b Key Laboratory of Advanced Technologies of Materials Ministry of Education, School of Materials Science and Engineering, Southwest Jiaotong University, Chengdu, 610031, China; c Department of Anorectal, The Thrid People's Hospital of Chengdu, Chengdu, Sichuan, China; d The School of Biomedical Engineering, Faculty of Engineering, The University of Sydney, Australia

**Keywords:** Pueraria, Traditional Chinese medicine, Plasma, Drug-eluting vascular stent coating, Atherosclerotic cardiovascular

## Abstract

To address the clinical challenges of stent thrombosis and restenosis in atherosclerosis intervention, this study developed a dual-crosslinked hydrogel coating (PSMASH—integrating methacrylated Pueraria polysaccharide (PSMA) and thiolated derivative (PSSH) based on Pueraria lobata polysaccharide). We used an ion-assisted plasma polymerization (IAPP) technique to construct micro-nano structures and thiol-ene click reaction sites on the surface of 316L stainless steel stents. This approach enabled robust covalent integration between the coating and the substrate. Simultaneously, the dual-network architecture of the hydrogel coating (synergistic covalent and dynamic crosslinking) conferred enhanced mechanical stability and controlled degradation properties. PSMASH promoted endothelial cell recovery and modulated the pathological phenotype of smooth muscle cells under oxidative stress. This effect was mediated by the controlled release of Pueraria-derived flavonoids. This "herb-to-device" (HTD) strategy integrates traditional Chinese medicinal components with advanced interface engineering. It establishes a novel therapeutic platform for atherosclerosis intervention that offers superior mechanical compatibility and multi-target synergistic efficacy.

## Graphical abstract

**Figure:**
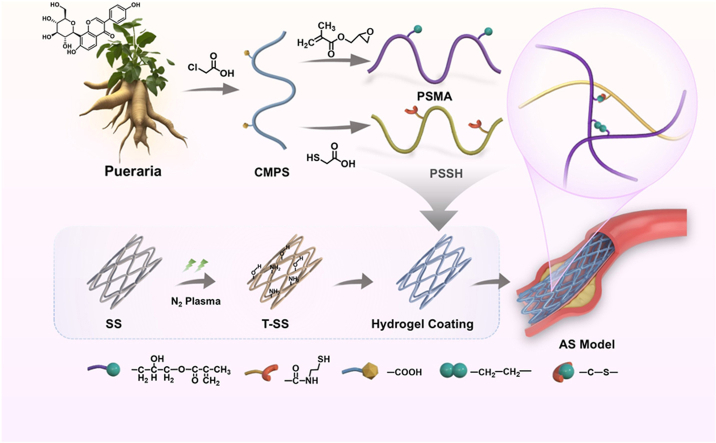

## 1 Introduction

In 2022, cardiovascular diseases (CVDs) affected 523 million individuals globally, accounting for 32 % of total worldwide mortality and representing the leading cause of death among non-communicable diseases [1]. Atherosclerosis, the primary pathological basis of CVDs [2], is directly implicated in 70 % of coronary artery disease (CAD) cases and 50 % of strokes [3]. Over the past four decades, the advent of vascular stenting technology—particularly the widespread application of drug-eluting stents (DES)—has significantly improved the survival rates and quality of life for CAD patients [4], Nevertheless, the complex pathophysiological microenvironment of atherosclerosis, characterized by chronic inflammation [5]、oxidative stress [6] and dysregulated lipid metabolism [7], remains inadequately addressed. This deficiency contributes to persistent post-stent complications, including delayed endothelialization and in-stent restenosis [8,9].

Current research has explored novel stent coating strategies, including a bioinspired nitric oxide (NO)-releasing endothelium-mimicking coating [10]and electrospun microchannel networks that guide oriented endothelial regeneration [11], Hydrogels have emerged as promising drug-delivery coatings due to their tissue-like mechanical properties, exceptional biocompatibility, biodegradability, and high drug-loading capacity [12]. For instance, extracellular matrix (ECM)-inspired hydrogel coatings composed of hyaluronic acid and collagen effectively enable sustained drug release [13]. Despite extensive investigation of such natural polymers in cardiovascular applications, their functionality remains focused on singular pathological aspects [14]. Moreover, physical drug encapsulation or simplistic crosslinking architectures face limitations including uncontrolled release kinetics, inflammatory responses, and mechanical mismatch [15], the uncontrolled "burst release" from hydrogels rapidly depletes anti-proliferative drugs, whose initial high dose impairs endothelial healing [16,17]. Subsequent drug absence fails to suppress vascular smooth muscle proliferation, directly leading to restenosis rates of 10–20 % [18,19]. Additionally, weak interfacial adhesion between stent substrates (e.g., metals, polymers) and hydrogels—attributed to low surface energy and chemical incompatibility—compromises coating integrity during balloon expansion in angioplasty, significantly increasing the risk of late stent thrombosis [20,21].

In recent years, puerarin (a major isoflavone from Pueraria lobata) has demonstrated multi-pharmacological activities—including vasodilation, endothelial function improvement, and lipid metabolism regulation—providing a foundation for clinical applications [22,23]. Pueraria polysaccharide (PLP), a natural carrier for puerarin and other isoflavonoids, exhibits a β-1,4-glycosidic bond structure analogous to vascular basement membrane components [24], This structural homology enables PLP to mimic native ECM, thereby promoting functional endothelial regeneration [25,26]. Concurrently, as a green renewable plant resource, PLP offers ecological sustainability. Crucially, PLP transcends its role as a passive structural scaffold. It acts as a natural reservoir for bioactive isoflavonoids like puerarin, facilitating the localized delivery of compounds with vasodilatory [27], antioxidant [28], and lipid-regulating effects [29]. This inherent multifunctionality represents a key advantage over conventional inert polysaccharides, including alginate and chitosan. These properties establish PLP-based materials as versatile polymeric platforms for delivering multi-therapeutic isoflavonoids in cardiovascular interventions.

Surface biofunctionalization of stents with natural polymers offers opportunities for regenerative medicine [13], However, conventional interfacial strategies—such as phenolic chemical grafting exemplified by polydopamine (PDA) and metal-polyphenol coordination networks—typically require alkaline conditions, while their degradation products (e.g., quinones) significantly compromise coating stability [[30], [31], [32]].

Ion-assisted plasma polymerization (IAPP) is a novel plasma-based surface modification technique that integrates plasma polymerization (PP) with plasma immersion ion implantation (PIII) [33,34]. In this process, the substrate surface is bombarded by highly energetic plasma species, producing a coating rich in surface-embedded free radicals. These reactive sites enable the covalent attachment of diverse bioactive molecules and particles [35], and the coating has demonstrated strong mechanical stability when applied to cardiovascular stents [36].

This study developed a biomimetic hydrogel coating for vascular stents. By employing IAPP technology, dense thiol-ene click-reactive sites were created on the surface of 316L stainless steel [37], additionally, micro-nano roughness was introduced to enhance mechanical interlocking. This approach enabled robust covalent integration of the dual-crosslinked PLP hydrogel network (PSMASH). Notably, this design features dual innovations. First, stress concentration in vessel walls is reduced by the PLP-based hydrogel's ability to mimic the mechanical properties of native ECM [38]. Second, plasma-induced molecular-level interface reconstruction confers exceptional adhesive strength to the coating while mitigating delamination risks during balloon expansion [39]. Through temporally controlled release of isoflavonoids, synergistic promotion of endothelial repair, lipid metabolism regulation, and phenotypic modulation is achieved, establishing an advanced therapeutic platform with integrated biofunctionality, mechanical compatibility, and treatment precision for atherosclerotic interventions.

## 2 Results and discussion

### 2.1 Design, synthesis, and material characterization of hydrogel coatings

**Fig. 1:**
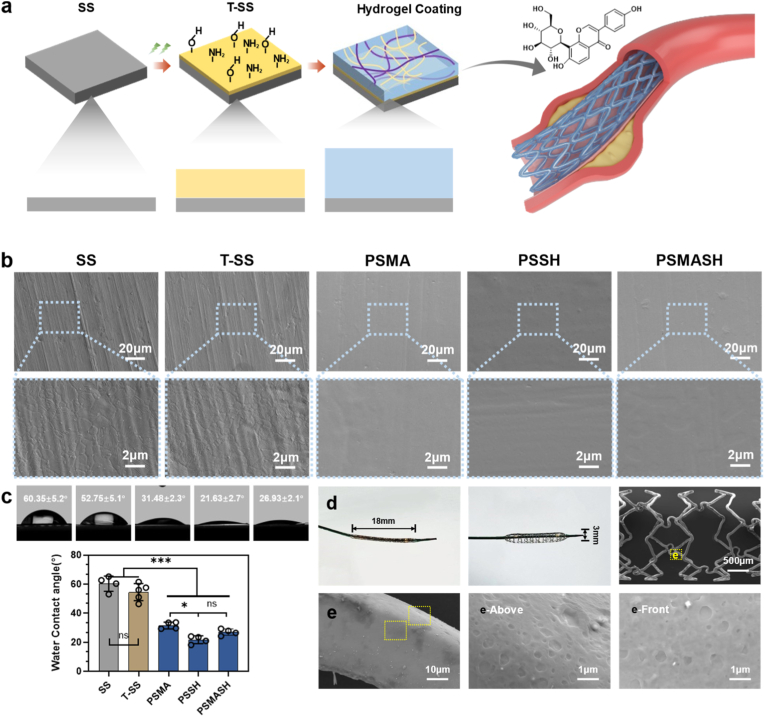
Preparation and Characterization of SS, T-SS, and PSMASH Hydrogel Coatings. (a) Schematic design of the coating architecture. (b) Surface morphology characterized by SEM (scale bar: 2 μm). (c) Water contact angles on coated surfaces. (d, e) Macroscopic images and corresponding SEM micrographs of simulated stents subjected to balloon compression and expansion cycles (scale bars: d = 500 μm, e = 10 μm).

Following surface modification of stainless-steel substrates via IAPP, radical-polymerizable hydrogel precursor solutions were applied using a single-step dip-coating method, enabling in situ gelation to form PSMA (single-crosslinked), PSSH (single-crosslinked), and PSMASH (dual-crosslinked) hydrogel coatings (Fig. 1a). This method is highly efficient and compatible with aqueous precursors, enabling conformal coating of the stent's complex 3D geometry. By ensuring intimate contact between the IAPP-activated surface and the precursors, it facilitates subsequent in-situ radical-induced cross-linking that covalently grafts the hydrogel network to the SS [40]. Fourier transform infrared (FTIR) spectroscopy (Nicolet iS20, Thermo Fisher Scientific, USA) analyses of pristine PLP, carboxylated (CMPS), vinyl-functionalized (PSMA), and thiolated (PSSH) derivatives—along with dip-coated hydrogel layers—revealed distinct chemical modifications (Fig. S1). Unmodified PS exhibited broad O-H stretching vibrations at 3200-3400 cm^−1^ [41]. CMPS showed characteristic carboxylate (COO^−^) absorption peaks at 1645, 1425, and 1325 cm^−1^ [42], with peak sharpness intensifying with higher degrees of substitution (DS), confirming successful carboxyl methylation. PSMA displayed diagnostic vinyl group signatures:=C-H stretching at 3020 cm^−1^, CH_2_ symmetric stretching at 2990 cm^−1^, C=C stretching at 1620-1700 cm^−1^, and = C-H bending at 714 cm^−1^ [43]. PSSH revealed weak -SH stretching (2500-2600 cm^−1^) [44,45], C-N stretching of secondary amides (1240 cm^−1^), N-H bending (1600-1640 cm^−1^), and characteristic secondary amide absorption at 1400 cm^−1^, verifying thioacetic acid conjugation via amination. Nuclear magnetic resonance (NMR) analysis (Fig. S2) further quantified vinyl functionalization in PSMA, with characteristic peaks at *δ* 6.4 ppm [-C(=O)O-R] and *δ* 2.0 ppm (CH_3_) indicating a DS of ∼20 %. Modified Pueraria polysaccharides demonstrated pronounced photoinitiator responsiveness. Under 365 nm UV irradiation, PSMA and PSMASH formulations underwent rapid cross-linking to form hydrogels, whereas PSSH failed to generate stable colloidal structures via photoinitiator-induced radical polymerization (Fig. S3a), The colloidal structure and crosslinking density of PSMASH are significantly higher than those of the single-crosslinked groups (80.2 % ± 2.67 %) (Fig. S14b). FTIR analysis of cross-linked structures revealed, PSMA coatings exhibited significantly attenuated C=C stretching vibrations at ∼1729 cm^−1^ with concomitant emergence of C-C bond absorption at 1126 cm^−1^ [46], confirming double-bond participation in polymerization. PSSH coatings showed complete disappearance of the broad -SH stretching band (2500-2600 cm^−1^) and displayed S-S stretching vibrations at 400-500 cm^−1^, indicating disulfide bond formation via radical coupling [44,47]. PSMASH spectra exhibited multiple characteristic peaks, revealing a dual-network architecture comprising covalent cross-links (C-C bonds) and dynamic thioester linkages (C-S bonds) formed through Michael addition between methacrylate and thiol groups [48]. Lyophilized cross-sections analyzed by scanning electron microscopy (SEM) demonstrated that PSMASH hydrogels possessed higher cross-linking density and more junction points (Fig. S3b), suggesting enhanced mechanical properties and biocompatibility.

Microstructural characterization was performed using field emission scanning electron microscopy (FESEM) on 316L stainless steel (SS), plasma-modified SS (T-SS), and single/dual-crosslinked hydrogel coatings (PSMA, PSSH, PSMASH). Figu e1b reveals complex textures and inherent defects on untreated SS and T-SS substrates. At 10,000 × magnification, SS surfaces exhibited typical rolled-metal characteristics: parallel streaks along the rolling direction and randomly distributed micro pits (1–5 μm diameter), with uniformly distributed grains and distinct grain boundaries. Notably, IAPP treatment significantly increased surface roughness, attributable to synergistic sputtering-etching and redeposition effects of high-energy plasma particles that induced atomic rearrangement. SEM images showed enhanced micro/nano-features, such as protrusions, nanoparticles, and nanopores. These structures potentially increase surface area/energy, thereby improving wettability and adhesion.

Following hydrogel coating, all samples displayed uniform thin-film structures, indicating strong interfacial bonding and homogeneous substrate coverage. This homogeneous interface enhances wettability and long-term stability, facilitating better bio integration while reducing risks of inflammation and thrombosis. Ellipsometry measurements (Fig. S4) further demonstrated that PSMASH coatings possessed the most uniform morphology with minimal defects, exhibiting an average thickness of 44.8 ± 2.59 nm–50 % greater than PSSH systems. Subsequently, we evaluated the wetting behavior of the coatings. Compared with pristine SS, T-SS substrates exhibited a modest reduction in water contact angle (WCA), consistent with plasma-induced micro- and nano-roughness amplifying Wenzel-state wetting [49]. In stark contrast, the hydrogel-coated groups (PSMA, PSSH, and PSMASH) achieved a pronounced WCA decrease by establishing continuous hydration shells and suppressing phase separation [[50], [51], [52]]. (Fig. 1c), crucial for reducing platelet adhesion, inhibiting thrombosis, and promoting endothelialization.

The hydrogel coating was immobilized on IAPP-modified vascular stents via a single-step dip-coating process. Adhesion strength was validated through simulated balloon compression and expansion cycles. SEM analysis revealed that PSMASH coatings maintained robust adhesion and sufficient flexibility to accommodate stent deformation during balloon inflation without cracking or delamination from stent struts (Fig. 1d and e). These results demonstrate exceptional interfacial bonding strength and deformability of PSMASH hydrogels on 316L stainless steel substrates, ensuring structural integrity during stent deployment and supporting their utility for vascular stent surface modification.

### 2.2 Stability of hydrogel coatings

**Fig. 2:**
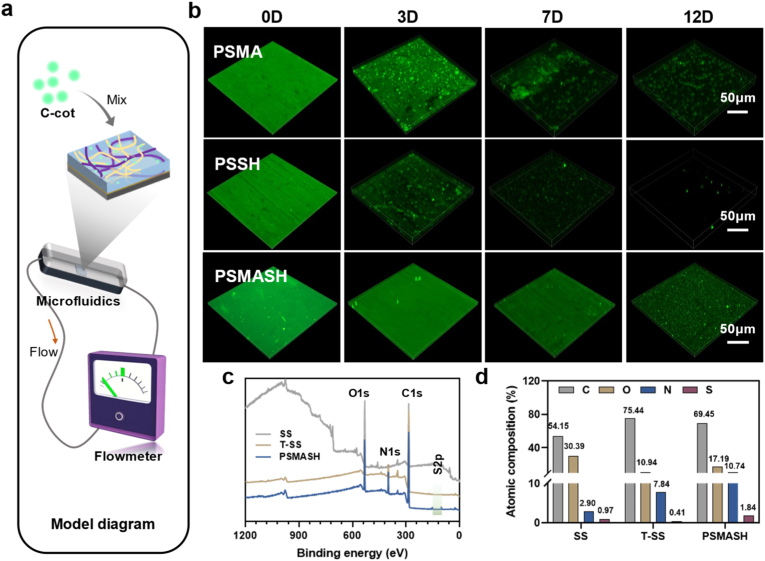
Stability and Degradation of Hydrogel Coatings. (a) Schematic of microfluidic shear simulation (PBS flow rate: 51.21 mL/min). (b) Confocal microscopy images showing coating retention (green fluorescence) at days 0, 3, 7, and 12. Scale bars: 50 μm. (c) XPS wide-scan spectra of coatings. (d) Elemental composition quantification (atomic %). (For interpretation of the references to colour in this figure legend, the reader is referred to the Web version of this article.)

Leveraging the superior encapsulation capacity of hydrogels and the long-term autofluorescence of carbon dots (CDs), CDs (100 μg/mL) were incorporated into the hydrogel precursor solution. During radical polymerization-mediated doping, CDs became embedded within the hydrogel matrix, enabling green-light-excitable fluorescence. A microfluidic chamber chip simulated high-shear blood flow conditions (PBS flow rate: 51.21 mL/min, 10 × physiological velocity) to assess coating stability and degradation (Fig. 2a). Confocal laser scanning microscopy (CLSM) performed on days 0, 3, 7, and 12 revealed coating retention proportional to fluorescence intensity (Fig. 2b). PSSH coatings exhibited significantly reduced fluorescence by day 3 and heterogeneous morphology with reduced structural integrity by day 7, attributable to their thin profile and low cross-linking density. In contrast, PSMASH dual-crosslinked coatings maintained dense, homogeneous films over 12 days. This robust stability, combined with a controlled degradation profile (Dominated by physical erosion by hydrodynamic shear and biochemical hydrolysis of PLP polymer chains) demonstrated in simulated body fluid (Fig. S16a), that aligns with the endothelialization timeline, ensures the coating can provide sustained mechanical support and drug release until vascular repair is complete.

Post-stent implantation, rapid endothelialization is critical. Coating degradation kinetics must synchronize with vascular healing, where ideal coatings gradually degrade without impeding endothelial coverage to ensure long-term vascular stability. X-ray photoelectron spectroscopy (XPS) quantified elemental composition, chemical states, and molecular structures of plasma-treated and hydrogel-coated surfaces. Wide-scan spectra revealed significantly increased nitrogen (N) content on plasma-modified stainless steel (10.74 % vs. untreated control), attributable to nitrogen radical bombardment that forms organic nitrogen moieties (e.g., -NH_2_, -CONH-) and nitrides (Fig. 2c and d). Following hydrogel coating, sulfur (S) content increased to 1.84 %, consistent with thioacetic acid incorporation in the dual-network architecture. High-resolution spectra (Fig. S5) further elucidated chemical states: C1s: Peaks at 284.8 eV (C-C/C-H/unreacted C=C in PSMA) and 286.0 eV (C-S bonds from thiol-ene click reactions). Binding energy shifts in PSMASH coatings suggested metal-sulfur interactions. N1s: Peaks between 399 and 401 eV confirmed nitrogen plasma grafting. Enhanced intensity in dual-network coatings indicated additional N from PSSH components. S2p: New peaks at 162.0 eV (thiolate/metal-sulfide bonds) and 163.5 eV (C-S bonds), with lower binding energies indicating covalent S-H/C-S bonding. The 168.2 eV peak (S-O bonds) validated hierarchical crosslinking. Thanks to Science Compass for providing invaluable assistance with the XPS test (www.shiyanjia.com).

The storage modulus (G′) and loss modulus (G″) of hydrogel patches were measured using a rheometer (Fig. S13). The results demonstrated that the PSMASH hydrogel exhibits viscoelastic properties closely resembling those of native cardiac ECM, with a storage modulus (G′) of approximately 2800 Pa and a loss modulus (G″) of 180 Pa(Fig. S15). These values are very similar to the native storage modulus of cardiac tissue layers (approximately 2.5 kPa) [53].

### 2.3 Hemocompatibility of Hydrogel Coatings

**Fig. 3:**
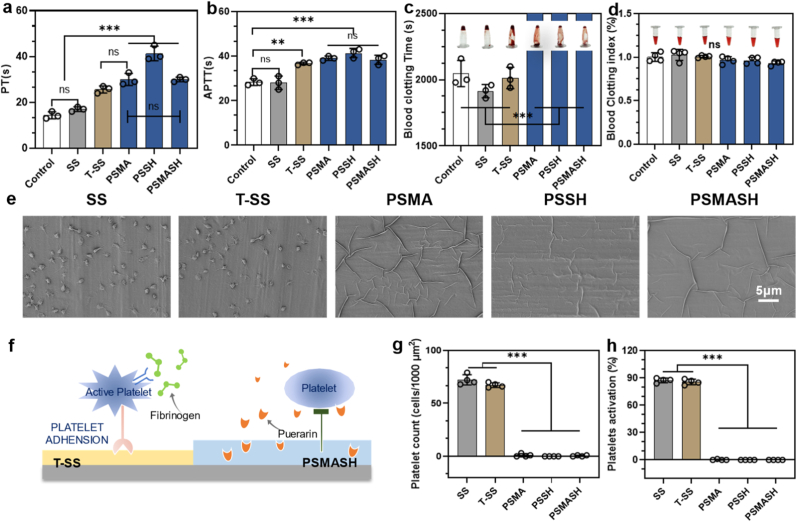
Hemocompatibility of Hydrogel Coatings. (a) Activated partial thromboplastin time (APTT). (b) Thrombin time (TT). (c) Whole blood coagulation time (BCT). (d) Blood coagulation index (BCI). (e) SEM images of platelet adhesion/activation on surfaces. Scale bars: 5 μm. (f) Schematic of anticoagulation mechanism via puerarin-mediated inhibition. (g) Quantitative analysis of platelet adhesion (cells/mm^2^). (h) Activated platelet percentage (n = 3); ∗p < 0.05, ∗∗p < 0.01, ∗∗∗p < 0.001.

Direct blood contact necessitates superior hemocompatibility for biomedical functionality. In prothrombin time (PT) and activated partial thromboplastin time (APTT) assays (Fig. 3a and b), PSMA, PSSH, and PSMASH coatings significantly prolonged coagulation times compared to unmodified SS and T-SS controls, indicating suppression of contact activation factors (e.g., FXII, FXI) in the intrinsic pathway. The modest APTT extension may reflect puerarin's selective targeting mechanisms. Plasma coagulation kinetics Fig. S6 demonstrated that PSMA and PSSH coatings reduced coagulation rates to near-zero slopes (0.012 ± 0.003 AU/min), comparable to low-molecular-weight heparin—suggesting potent anticoagulant effects. This phenomenon correlates with puerarin's ability to inhibit tissue factor (TF)-triggered coagulation feedback loops. All coatings maintained erythrocyte membrane integrity, with hemolysis rates <5 % (peak: 0.45 %), complying with ISO 10993-4 standards (Fig. S7).

Whole blood coagulation time (BCT) and blood coagulation index (BCI) analyses confirmed significant anticoagulant properties (>40 min BCT) for all hydrogel groups (Fig. 3c). Mechanistically, puerarin inhibits key coagulation cascade steps, including platelet aggregation and thrombus formation. Consistently low BCI values (Fig. 3d) confirmed minimal clot formation on coated surfaces.

After incubating samples with platelet-rich plasma (PRP), scanning electron microscopy (SEM) revealed pronounced platelet adhesion and activation on SS and T-SS surfaces (Fig. 3e–g, h), likely attributable to their physicochemical properties that promote platelet aggregation. In contrast, PSMA, PSSH, and PSMASH hydrogel coatings exhibited no platelet adhesion, demonstrating superior antiplatelet properties. This performance stems primarily from the coatings' hydrophilicity and low surface roughness, which facilitate a hydration layer that prevents direct platelet-material contact. Mechanistically, puerarin—a key component—acts as a natural anticoagulant by binding to platelet membrane glycoprotein receptors, thereby inhibiting interactions with plasma proteins and suppressing platelet aggregation/activation (Fig. 3f) [[54], [55], [56]].

**Fig. 4:**
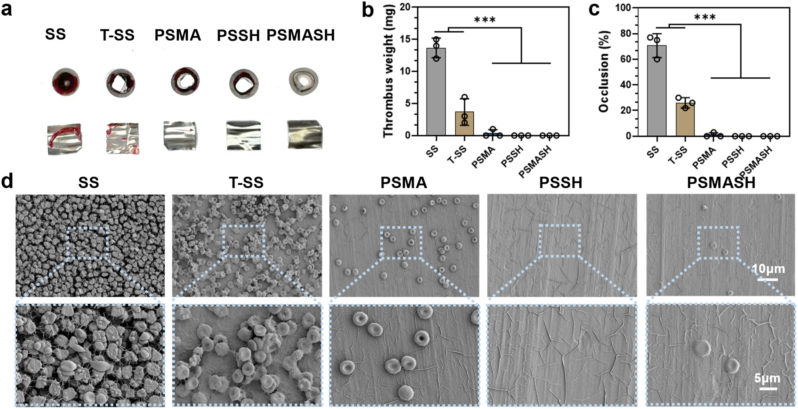
Dynamic Hemocompatibility Evaluation. (a) Cross-sectional schematic of sample foil catheter post-ECC and surface-adhered thrombi. Red arrows indicate flow direction. (b) Thrombus weight on sample surfaces (mg). (c) Occlusion rate quantification (%). (d) SEM micrographs of surface-adherent components. (n = 3); ∗∗∗p < 0.001. (For interpretation of the references to colour in this figure legend, the reader is referred to the Web version of this article.)

An extracorporeal circulation (ECC) model in New Zealand rabbits was established to evaluate the performance of PSMA, PSSH, and PSMASH hydrogel coatings in a semi-in vivo system. Following 1-h ECC operation, occlusion rates were calculated by measuring luminal cross-sectional areas pre- and post-circulation. Luminal images (Fig. 4a) revealed significantly reduced occlusion in T-SS versus bare SS controls, while hydrogel coatings exhibited negligible occlusion, further confirming superior hemocompatibility. Microstructural analysis (Fig. 4d) demonstrated extensive blood cell adhesion, fibrin networks, and thrombus formation on SS surfaces, whereas hydrogel-coated surfaces maintained intact erythrocytes with characteristic biconcave discoid morphology and no platelet adhesion/activation. These results indicate effective suppression of platelet activation and thrombogenesis by the coatings. Quantitative analyses of thrombus weight Fig. 4b and c confirmed exceptional anticoagulant properties of PLP hydrogels. The low occlusion rates (≤3.2 %) and minimal thrombus formation (1.8–2.3 mg) suggest significantly reduced coagulation risks, meeting clinical requirements for blood-contacting materials with substantial translational potential [57,58].

### 2.4 The cell compatibility and functionality of the hydrogel coating

The integrity and regenerative capacity of the endothelial cell (EC) layer are critical for maintaining vascular homeostasis. Rapid endothelial coverage reduces platelet aggregation, inflammation, thrombotic risks, and neointimal hyperplasia, thereby mitigating restenosis. In vitro experiments confirmed favorable EC adhesion and proliferation on all hydrogel coatings. Compared to SS and T-SS controls, hydrogel-modified surfaces exhibited significantly higher EC coverage (p < 0.01) with characteristic cobblestone morphology (Fig. S8a、S9a). Enhanced cell viability at day 3 suggests cytoprotective and proliferative effects from bioactive PLP, demonstrating improved biocompatibility and pro-endothelial regeneration. Conversely, hydrogel-released isoflavonoids (e.g., puerarin) effectively inhibited vascular smooth muscle cell (SMC) proliferation and induced apoptosis. Mechanistically, they disrupted cytoskeletal reorganization (F-actin disassembly) and downregulated motility-related proteins [59,60] (e.g., RhoA, FAK), explaining apoptotic morphology in SMC-coating co-cultures at day 3, CCK-8 assays corroborated significantly reduced SMC viability in hydrogel groups (p < 0.001 vs. control), with no statistical difference between PSSH and PSMASH (Fig. S9b). Trans well migration assays further revealed potent inhibition of SMC migration by PSMA, PSSH, and PSMASH coatings (Fig. 6b、S10).

### 2.5 Protective effects of hydrogel coatings on cardiovascular cells under oxidative stress

Atherosclerotic (AS) microenvironments and stent implantation disrupt vascular homeostasis, with core pathological mechanisms encompassing dyslipidemia, chronic inflammation, endothelial dysfunction, and aberrant vascular smooth muscle cell (VSMC) proliferation/migration. During AS progression, key cellular events—macrophage foam cell formation, endothelial injury, and VSMC phenotypic switching—collectively drive plaque development and vascular stenosis. Based on prior experimental evidence (Fig. S11), an oxidative stress model was established by treating cells with 50 μg/mL oxidized low-density lipoprotein (ox-LDL) for 24 h, simulating AS-related cellular damage and foam cell transformation. This model validated the regulatory effects of PLP hydrogel coatings on Lipid metabolism homeostasis、Inflammatory response modulation、Proliferation activity normalization and Phenotypic transition control.

**Fig. 5:**
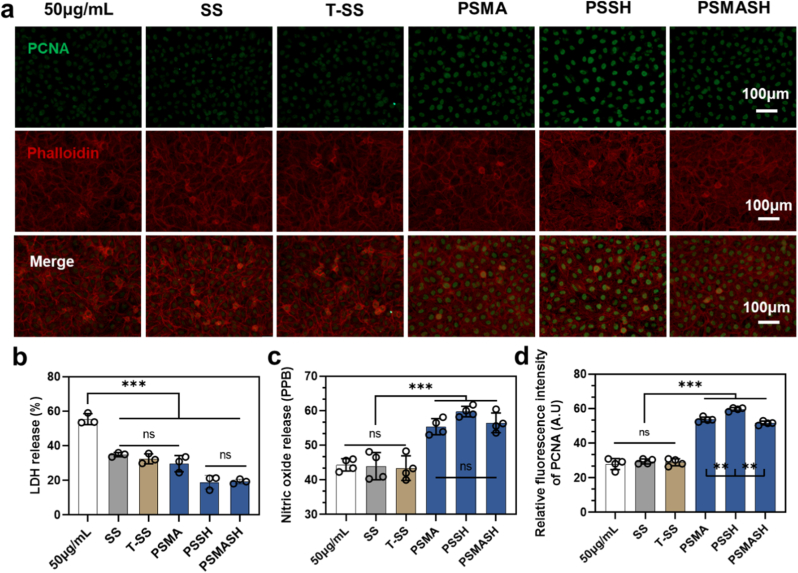
Endothelial Cell (EC) Status on Hydrogel Coatings under Oxidative Stress. (a) Fluorescent staining of ECs: Green: PCNA (proliferating cell nuclear antigen, nuclei) Red: Phalloidin (F-actin cytoskeleton) (b) Lactate dehydrogenase (LDH) release from ECs at 24 h. (c) Nitric oxide (NO) production by ECs at 24 h. (d) Semi-quantitative analysis of PCNA^+^ nuclei fluorescence intensity. Data: mean ± SEM (n = 4 independent experiments); ∗p < 0.05, ∗∗p < 0.01, ∗∗∗p < 0.001 vs. ox-LDL group. Scale bars: 1000 μm. (For interpretation of the references to colour in this figure legend, the reader is referred to the Web version of this article.)

Under oxidative stress, hydrogel coatings significantly reduced lactate dehydrogenase (LDH) release compared to the ox-LDL model group (Fig. 5b), The PSMASH group maintained 89 % ± 5 % cell viability—significantly higher than the model group's 54 % ± 7 % (p < 0.001)—with concurrent restoration of PCNA expression (Fig. 5d), demonstrating potent endothelial cytoprotection. Notably, NO production in coated groups increased 2.5-fold versus the model group (Fig. 5c), Puerarin released from the PSMASH coating activates estrogen receptor-mediated PI3K/Akt and CaMKII/AMPK pathways [[61], [62], [63]]. This stimulates eNOS phosphorylation and NO production, alleviating ox-LDL-induced endothelial dysfunction. Potential upregulation of VEGF signaling and integrin-mediated cell adhesion further support this effect synergistically [64].

**Fig. 6:**
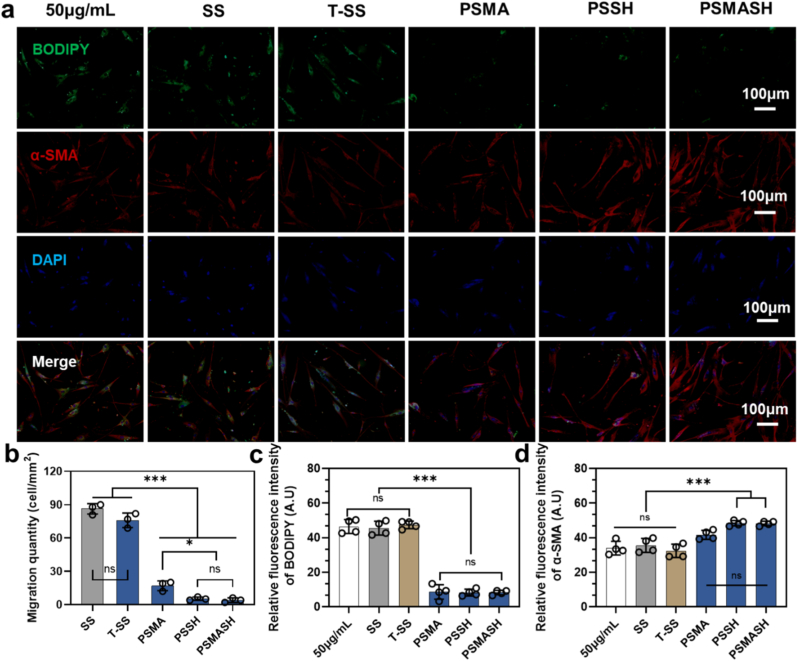
Vascular Smooth Muscle Cell (VSMC) Status under Oxidative Stress. (a) Dual fluorescence staining:Green: BODIPY® 493/503 (intracellular neutral lipids), Red: α-smooth muscle actin (α-SMA, contractile phenotype marker). (b) Transwell migration assay quantification (migrated VSMCs/field). (c) Semi-quantitative analysis of BODIPY fluorescence intensity (lipid accumulation). (d) Semi-quantitative analysis of α-SMA^+^ area percentage (contractile phenotype maintenance). Scale bars: 100 μm (a).∗p < 0.05, ∗∗p < 0.01, ∗∗∗p < 0.001. (For interpretation of the references to colour in this figure legend, the reader is referred to the Web version of this article.)

In the vascular smooth muscle cell (VSMC) model, the ox-LDL group exhibited significantly increased BODIPY fluorescence intensity, indicating lipid droplet accumulation (Fig. 6a). Conversely, hydrogel-coated groups showed reduced green fluorescence (neutral lipids) with enhanced contractile marker fluorescence, demonstrating substantially decreased lipid deposition. The PSMASH coating group achieved 78 % ± 6 % α-SMA^+^ area coverage—a 2.1-fold increase versus the model group (Fig. 6c and d). Mechanistically, this effect is attributable to Pueraria-derived flavonoids, particularly puerarin, which suppresses foam cell formation by inhibiting the p38 MAPK and JNK signaling pathways to attenuate OX-LDL-induced VSMC proliferation [65,66], and modulates phenotypic switching via the PI3K/Akt/mTOR axis [67]. This effect, attributable to Pueraria-derived flavonoids, suppresses foam cell formation and may significantly reduce atherosclerotic plaque development and restenosis risk.

### 2.6 Atherosclerotic (AS) animal model establishment and in vivo stent evaluation

All animal procedures were approved by the Animal Ethics Committee of Southwest Jiaotong University (SWJTU-2208-SCS(070) and complied with NIH Guidelines for the Care and Use of Laboratory Animals. Fig. S12 summarizes key model characteristics. Compared to the normal group, the model group exhibited significantly elevated plasma lipid levels: triglycerides (TG) increased to 2.28 ± 0.5 mmol/L, total cholesterol (TC) to 32.24 ± 0.6 mmol/L, low-density lipoprotein cholesterol (LDL-C) to 21.13 ± 3.5 mmol/L, and high-density lipoprotein cholesterol (HDL-C) to 1.86 ± 0.2 mmol/L, which may be attributed to the substantial rise in TC concentration. Fasting blood glucose levels markedly increased to 14.24 ± 3.5 mmol/L, exceeding diagnostic thresholds for diabetes, accompanied by substantial weight gain, indicating a hyperglycemic and hyperlipidemic state. Histopathological analysis of embedded and frozen sections revealed characteristic atherosclerotic pathological alterations: significant carotid intimal thickening (intima-to-media thickness ratio increased 2.8 ± 0.4-fold), fibrous cap formation on plaque surfaces, and 67.1 % ± 7.8 % reduction in luminal area. Oil Red O staining demonstrated lipid cores occupying 58.4 % ± 7.0 % of plaque area, with abundant cholesterol clefts and clusters of foam cells containing cytoplasmic lipid droplets (diameter: 0.2–2 μm; vacuolated cells >40 %). Medial thinning featured reduced or absent smooth muscle cells, scattered microcalcifications or focal calcification, while adventitial remodeling included neovascularization, fibroconnective tissue proliferation, and inflammatory cell infiltration.

**Fig. 7:**
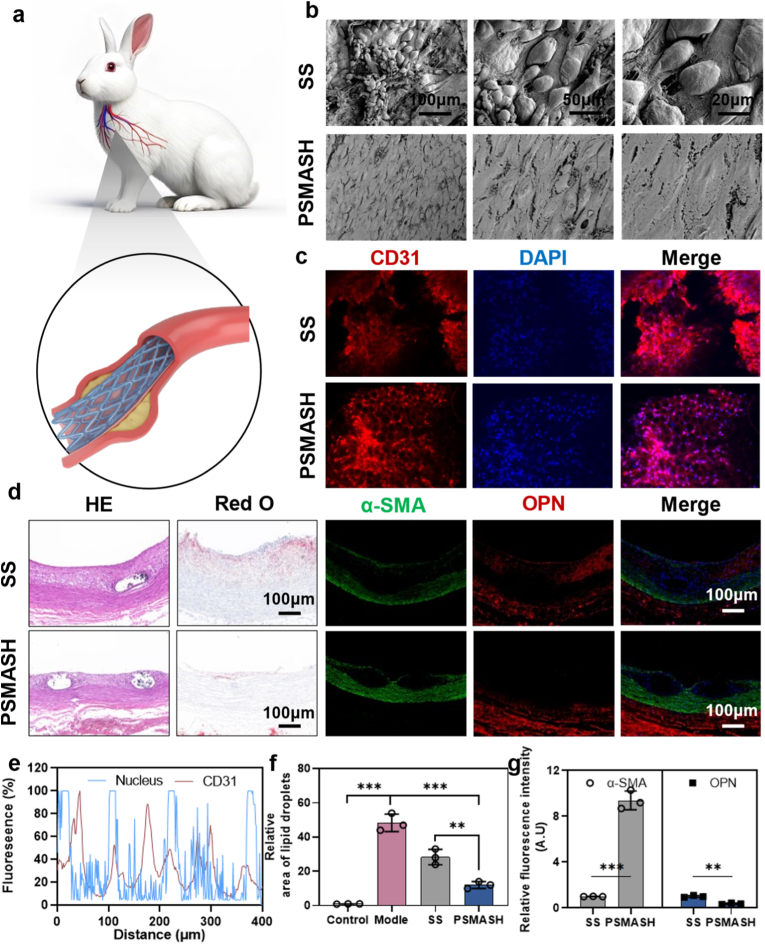
Histological Analysis of Hydrogel-Coated Stents in Aortic Plaques of AS Model Rabbits at 7D/30D Post-Implantation. (a) Stent deployment schematic. (b) SEM surface morphology of neointima at 7D. Scale bar: 200 μm. (c) CD31 immunofluorescence of vascular tissue surface at 7D. Scale bar: 100 μm. (d) Serial staining: H&E, Oil Red O, CD31, α-SMA, osteopontin (OPN). Scale bars: H&E/Oil Red O = 500 μm; immunofluorescence = 50 μm. (e) Pearson correlation analysis of CD31^+^ endothelial coverage. (f) Quantitative analysis of Oil Red O-positive plaque area (%). (g) Fluorescence quantification of SMC phenotypic markers (α-SMA^+^/OPN^−^). (n = 4); ∗p < 0.05, ∗∗p < 0.01, ∗∗∗p < 0.001. (For interpretation of the references to colour in this figure legend, the reader is referred to the Web version of this article.)

Based on preceding results, SS stents and PSMASH-coated stents were implanted in AS animal models. Fig. 7a illustrates the stent deployment procedure. At 7 days post-implantation, SS stents were fully covered by randomly organized neointimal tissue Fig. 7b indicating heterogeneous cellular/extracellular matrix composition. In contrast, PSMASH-coated stents exhibited a confluent, highly aligned cell layer—characteristic of functional endothelium. CD31 immunofluorescence staining further confirmed a continuous endothelial monolayer on PSMASH surfaces (Fig. 7c) Subsequently, we used Pearson correlation analysis to quantitatively assess the degree of colocalization of CD31 signals (endothelial cells) and DAPI signals (nuclei) on the scaffold surface. Pearson correlation analysis demonstrated dense CD31^+^ coverage, validating enhanced endothelialization mediated by the hydrogel coating (Fig. 7e).

To analyze the composition and cellular phenotypes of peri-stent hyperplastic tissues, vascular sections were paraffin-embedded and subjected to multidimensional histological analysis 30 days post-implantation (Fig. 7d). H&E staining revealed significantly reduced neointimal thickness in the PSMASH group versus SS controls (p < 0.01), while Oil Red O quantification demonstrated a 67 % reduction in lipid core area within plaques (Fig. 7f), indicating effective suppression of lipid accumulation. Histochemical Staining experiments were completed with the assistance of Lilai Biotechnology Co., Ltd. (Chengdu, China). Puerarin as a multi-target regulator of lipid metabolism, directly target cellular lipid metabolism by reducing ox-LDL uptake [68] and promoting cholesterol efflux. [69]. Co-staining of α-SMA (contractile phenotype marker) and osteopontin (OPN, synthetic phenotype marker) assessed neointimal hyperplasia potential. PSMASH exhibited substantially higher α-SMA^+^ expression than SS stents, indicating promotion of contractile SMC phenotypes and consequently greater anti-hyperplastic potential. Conversely, SS controls showed abundant OPN^+^ cells correlating with progressive neointimal hyperplasia. Combined with in vivo SMC proliferation/migration and phenotypic assessments, these findings suggest PSMASH hydrogel coatings may maintain vascular patency by mitigating ox-LDL-induced SMC phenotypic switching. Collectively, the results demonstrate PSMASH coatings facilitate inflammation resolution, SMC phenotypic regulation, and endothelialization, exhibiting promise for achieving ideal vascular remodeling.

Future studies should use large animal models with extended observation periods (≥6 months) to assess endothelial maturation, late-term stent patency under physiological flow, coating biodegradation kinetics, and additionally, the adaptability of the coating in complex physiological environments, especially in calcified plaques or high-shear regions.

Furthermore, successful clinical translation necessitates scaling manufacturing under GMP conditions, ensuring batch consistency, and verifying long-term biocompatibility according to ISO 10993 standards. Establishing a clear regulatory pathway through early engagement with agencies is also critical for approval of this combination product. These steps are essential to fully realizing PSMASH's potential in improving outcomes for advanced atherosclerotic disease.

## 3 Discussion

This study successfully developed and validated a novel dual-crosslinked hydrogel coating (PSMASH) based on IAPP interfacial engineering and synergistic PLP, providing an innovative solution for biofunctional modification of vascular stents in atherosclerotic (AS) intervention. The IAPP technique created micro-nano structured interfaces with high reactivity, while the dual-network architecture—combining covalent C-C bonds and dynamic C-S bonds through photoinitiated thiol-ene click chemistry—provided exceptional mechanical stability and interfacial adhesion. This robust integration was confirmed by SEM analyses showing no delamination or cracking after simulated balloon expansion, indicating reliable anti-phase-separation capability during stent deployment. PSMASH exerts its therapeutic effects through a sophisticated dual-pathway mechanism mediated by sustained isofiavonoids release. The activation of PI3K/Akt/eNOS signaling promotes endothelial regeneration and NO production, enhancing vascular homeostasis and re-endothelialization. Concurrently, suppression of the RhoA/ROCK pathway inhibits VSMC proliferation, migration, and phenotypic switching, effectively disrupting the cycle of endothelial damage and hyperplasia that drives restenosis [59]. As evidenced in atherosclerosis (AS) rabbit models, this mechanism results in significantly reduced lumen occlusion (<5 %) and neointimal hyperplasia, while simultaneously promoting endothelial coverage. The concurrent reduction in lipid core area and modulation of α-SMA^+^ expression further confirm that PSMASH not only provides a physical barrier but also actively facilitates vascular homeostasis by steering the pathological microenvironment toward a stable phenotype.

This strategy fundamentally overcomes the inherent limitations of traditional Chinese medicine administered orally or by injection, such as high systemic exposure and first-pass effects [70,71]. By leveraging its dual-network hydrogel, the strategy achieves sustained drug release at the target site, effectively maintaining the local therapeutic concentration [72,73].

## 4 Conclusion

This study pioneers an herb-to-device materialization strategy transcending traditional oral/injectable delivery limitations of Chinese medicine. By integrating plant-derived bioactive compounds (PLP and isoflavonoids) with demonstrated cardiovascular protective effects into vascular implants via advanced materials science and interface engineering—including plasma activation and dual-crosslinked hydrogels—we established a precision-functionalized interventional platform. The IAPP-enabled robust interfacial integration and ECM-mimetic hydrogel viscoelasticity ensure mechanical adaptability and interfacial stability under vascular hemodynamics. Concurrently, the dual-network architecture provides an ideal drug-eluting platform, leveraging PLP's intrinsic biocompatibility as a natural carrier to achieve temporally controlled release of multiple therapeutics. This approach targets critical atherosclerosis (AS) pathological pathways synergistically. It promotes endothelial repair, normalizes lipid metabolism, and modulates vascular smooth muscle cell (SMC) phenotype. This avoids the burst release and systemic toxicity often seen with conventional drug-eluting coatings. This approach presents a promising device solution for precision intervention in atherosclerosis while establishing a paradigm for plant-derived bioactive in regenerative medicine and implantable devices, marking a convergence point of traditional medicine modernization and precision healthcare in cardiovascular innovation.

## CRediT authorship contribution statement

**Jingyue Wang:** Investigation, Data curation. **Ge Yin:** Methodology, Conceptualization. **Leila Mamizadeh Janghour:** Validation, Supervision. **Sheng Dai:** Formal analysis, Data curation, Conceptualization. **Behnam Akhavan:** Conceptualization, Funding acquisition. **Maolin Sun:** Conceptualization, Visualization. **Ansha Zhao:** Writing – review & editing, Validation, Supervision.

## Experimental section

The Experimental Section is available in the Supporting Information.

## Declaration of competing interest

There are no conflicts to declare.

## Data Availability

The data that has been used is confidential.
