# Supplementary material for: Plasma-reinforced dual-crosslinked Pueraria hydrogel coating for synergistic atherosclerosis intervention
Source: Mater Today Bio. 2025 Sep 14;35:102313. doi: 10.1016/j.mtbio.2025.102313 (PMC12481940; doi:10.1016/j.mtbio.2025.102313)
Supplement: Multimedia component 1 [file mmc1.docx]

**Supporting Information**

**Plasma-Reinforced Dual-Crosslinked Pueraria Hydrogel Coating for Synergistic Atherosclerosis Intervention**

Jingyue Wang^a,b,1^, Ge Yin^c,1^, Leila Mamizadeh Janghourr^d^, Sheng Dai^a^, Maolin Sun^a,b^, Ansha Zhao^a,b,*^

**Authors address:**

1. Institute of Biomedical Engineering, College of Medicine, Southwest Jiaotong University, Chengdu 610031, Sichuan, China
2. Key Laboratory of Advanced Technologies of Materials Ministry of Education, School of Materials Science and Engineering, Southwest Jiaotong University, Chengdu 610031, P. R. China.
3. Department of Anorectal, The Thrid People’s Hospital of Chengdu, Chengdu, Sichuan, China.
4. The School of Biomedical Engineering, Faculty of Engineering, The University of Sydney, Australia

***Corresponding author**

Name: Ansha Zhao

Address: Institute of Biomedical Engineering, College of Medicine, Southwest Jiaotong University, Chengdu 610031, Sichuan, China

E-mail: anshazhao@263.net


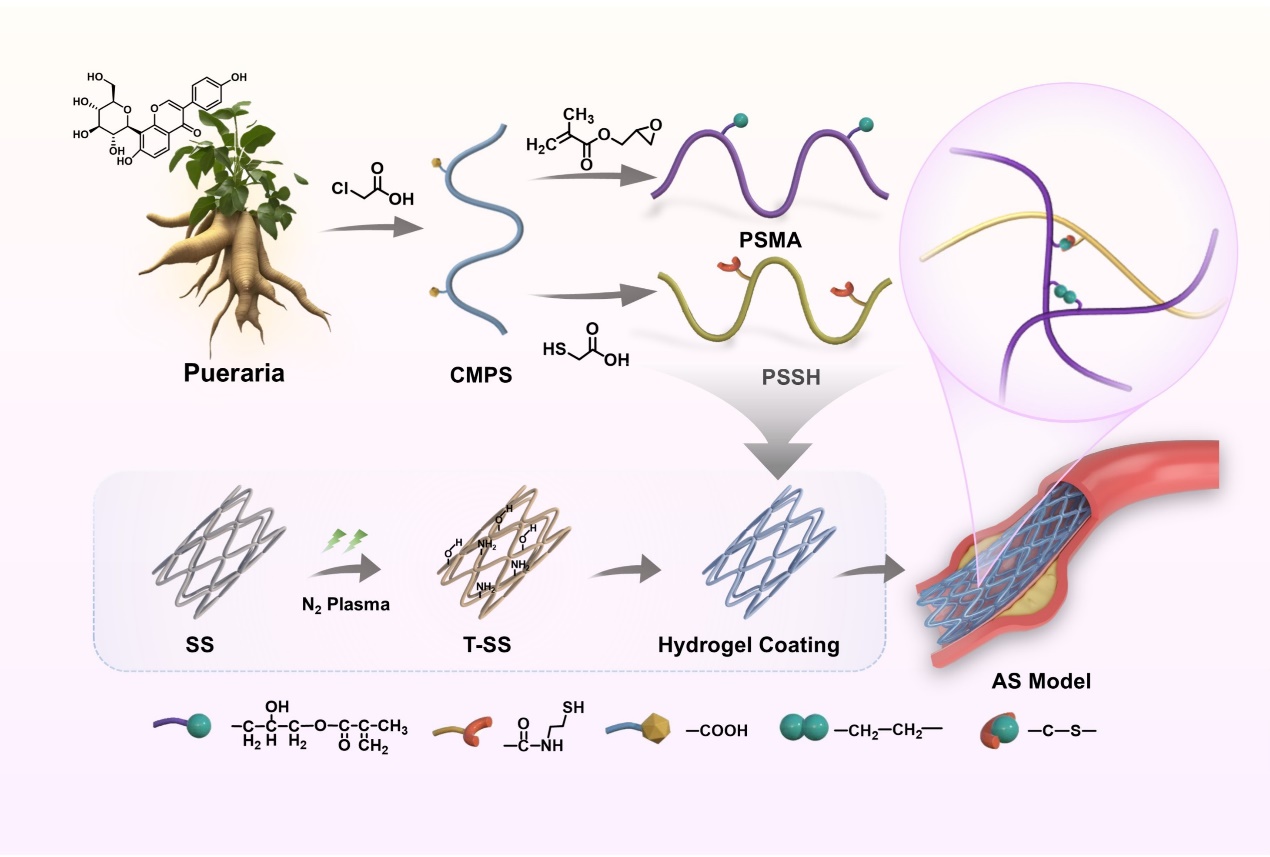
**Abstract figure**

**Materials**

Pueraria powder was procured from Jingmen (Hubei, China); 1-ethyl-(3-dimethylaminopropyl) carbodiimide hydrochloride (EDC) and N-hydroxysuccinimide (NHS) (purity ≥99.8%) from Shanghai Aladdin; glycidyl methacrylate (GMA) and thioglycolic acid from Beijing Solarbio; anhydrous ethanol, methanol (purity >99%), and concentrated hydrochloric acid from Chengdu Kelong Chemical; prothrombin time (PT) and activated partial thromboplastin time (APTT) assay kits from Shanghai Ruji Biotechnology; high-glucose DMEM, fetal bovine serum (FBS), SMCM medium (Sciencell, 1101), and trypsin from Gibco (USA); Oil Red O dye, TNF-α, and IL-10 ELISA kits from Shanghai Yeasen Biotechnology; Cell Counting Kit-8 (CCK-8) from Dojindo Molecular Technologies (Japan); lactate dehydrogenase cytotoxicity assay kit (LDH, HY-K1090) from MedChemExpress (MCE); Transwell chambers (PC, 8 μm) and Calcein-AM/PI live-dead stain from Servicebio; PCNA, α-SMA, and CD31 primary antibodies from Abcam; BODIPY 493/503 from Beyotime Biotechnology (Shanghai); New Zealand rabbits from Chengdu Dashuo Experimental Animal Co., Ltd. (with approval from the Institutional Animal Care and Use Committee); and high-fat high-sucrose rabbit diet from Beijing Ensiweier Co., Ltd.

**Characterizations**

The experimental equipment included an electronic balance (BSA224S, Sartorius), constant-temperature water bath (SHA-B, Wuhan Greamu), field-emission transmission electron microscope (TEM, JEOL JEM 2100F, Japan), field-emission scanning electron microscope (SEM, ZEISS Gemini 300, Germany), high-temperature autoclave (Shanghai Sanshen), 3D printer (microarch s140pro, SLA), X-ray photoelectron spectrometer (XPS, Thermo Scientific K-Alpha, USA), Malvern laser particle size analyzer (ZEN3690), electrothermal constant-temperature blast dryer (DHG-9070A, Shanghai Qixin), Fourier-transform infrared spectrometer (Nicolet iS20, Thermo), nuclear magnetic resonance spectrometer (MHz Bruker Avance NEO, Germany), ultra-high-performance liquid chromatograph (UHPLC, Agilent 1290 Infinity II, USA), constant-temperature cell incubator (Thermo, USA), microplate reader (Varioskan Lux, Thermo), fluorescence inverted microscope (Olympus, Japan), laser confocal microscope (Air+, Nikon, Japan), and slide scanner (DM6B, ZEISS, Germany).

**Experiment Section**

**Synthesis of Modified Pueraria Polysaccharides**

Carboxymethyl Pueraria polysaccharide (CMPS) was synthesized by alkalizing Pueraria powder with 10.8 g NaOH in ethanol for 1.5 h, followed by etherification with chloroacetic acid (CH₂ClCOOH) at 40°C for 4 h. The resulting mixture was filtered, precipitated in 80% ethanol, and neutralized to pH 7.0 using 2 mol/L HCl. The precipitate was washed 4–5 times with 80% ethanol, yielding pale-yellow solid lumps that were vacuum-dried at 55°C for 24 h. Methacrylated Pueraria polysaccharide (PSMA) was prepared via epoxy ring-opening reaction: 2 g CMPS dissolved in 200 mL reverse osmosis (RO) water was heated to 90°C in a water bath, acidified to pH 3.5 with HCl, then reacted with 5 mL glycidyl methacrylate (GMA) for 10 h to form ether bonds. Thiolated Pueraria polysaccharide (PSSH) was synthesized by grafting thioglycolic acid onto CMPS via amidation: 500 mg 1-ethyl-(3-dimethylaminopropyl) carbodiimide hydrochloride (EDC) and 500 mg N-hydroxysuccinimide (NHS) were added to the solution and stirred at room temperature for 2 h, followed by reaction with 2 g thioglycolic acid under dark conditions for 24 h.

**Phytochemical Profiling of Native Pueraria Polysaccharide**

High-performance liquid chromatography (HPLC) was performed on an Agilent 1290 Infinity II system (Agilent Technologies, USA) to characterize the constituents of native Pueraria powder. The powder was suspended in 95 % (v/v) ethanol and subjected to reflux extraction at 80 °C for 3 h. After rotary evaporation to dryness, the residue was reconstituted in methanol (1 g powder/10 mL solvent), filtered through a 0.22 μm membrane, and injected for analysis. Chromatographic separation was achieved on an Agilent Eclipse XDB-C18 column (4.6 mm × 250 mm, 5 μm) maintained at 30 °C. Mobile phase A was acetonitrile and mobile phase B was 0.1 % (v/v) phosphoric acid in water, delivered according to the following gradient: 0–5 min, 5 % A; 5–25 min, 5–25 % A; 25–35 min, 25–35 % A; 35–40 min, 35–5 % A; 40–45 min, 5 % A. The flow rate was 0.5 mL min⁻¹, and UV detection was set at 260 nm (reference wavelength 360 nm). Injection volume was 5 μL. Under these conditions, all analytes were baseline-separated with good resolution.

**IAPP Modification of Stainless Steel Surface**

Stainless steel (SS) sheets (0.2 mm thickness) were cut into 10×10 cm pieces, ultrasonically cleaned in acetone and ethanol (25°C, 20 min each), rinsed with Milli-Q water, and mounted on a sample holder. A cylindrical IAPP reactor (stainless steel) was connected to an ENI radiofrequency (RF) power supply (13.56 MHz). The substrate holder was coupled to a RUP-6 pulse generator (GBS Elektronik GmbH) to enhance ion bombardment via negative bias voltage. The vacuum system comprised an Ebara PDV250 mechanical pump and Edwards NEXT400 turbomolecular pump, connected to the chamber via a butterfly valve. Precursor gases were introduced through a showerhead assembly linked to Allicat Scientific mass flow controllers. The sample holder was electrically connected to a grid positioned 5 cm in front of it. After cleaning, the chamber was evacuated to 5×10⁻⁵ Torr, followed by introduction of deposition gases. A mixture of acetylene (C₂H₂), nitrogen (N₂), and argon (Ar) was fed at constant flow rates (5, 5, and 20 sccm, respectively). At a working pressure of 110 mTorr, a 50 W RF power and 500 V negative bias voltage were applied for 4 min, yielding plasma-modified tetrahedral carbon-coated stainless steel (T-SS).

**Preparation of PSMA, PSSH, and PSMASH Hydrogel Coatings**

PSMA single-crosslinked, PSSH single-crosslinked, and PSMASH dual-crosslinked hydrogel coatings were fabricated on T-SS substrates and plasma-modified cardiovascular stents using a simplified one-step dip-coating method. Samples were ultrasonically cleaned in ethanol and single-distilled RO water, dried, then immersed separately in 5 wt% PSMA solution, 5 wt% PSSH solution, or a 1:1 (v/v) mixture of PSMA and PSSH solutions at equal concentrations (5 wt%). Following 24 h reaction at 37°C, coatings were ultrasonically rinsed with RO water, dried under nitrogen flow, and stored in a vacuum drying oven.

**Characterization of Chemical Structure and Composition of Hydrogel Coatings**

Successful fabrication of PSMA and PSSH single-crosslinked hydrogel coatings and PSMASH dual-crosslinked hydrogel coatings was confirmed using Fourier-transform infrared spectroscopy (FTIR, Nicolet iS20, Thermo Fisher Scientific, USA) in attenuated total reflectance (ATR) mode, with spectra collected from 500 to 4000 cm⁻¹ at 4 cm⁻¹ resolution. X-ray photoelectron spectroscopy (XPS, Thermo Scientific K-Alpha) determined elemental composition and chemical states using monochromatic Al Kα radiation (1486.6 eV) at 12 kV and 6 mA, performing survey scans (150 eV pass energy, 1 eV step) and high-resolution scans (50 eV pass energy, 0.1 eV step) with C1s (284.8 eV) as reference. Surface morphology was characterized by scanning electron microscopy (SEM, JEOL JSM-7800F) after gold sputtering. Coating thickness, refractive index, and roughness were measured via spectroscopic ellipsometry (M-2000v, J.A. Woollam). The coating yield was determined by weighing with a 0.1 mg precision electronic balance, calculated using the formula:

$Coating Yield (\%) = (m\_coating / m\_total\_solid) \times100\%$(Equation S1)

where m_coating represents the mass of the dried coating, and m_total_solid denotes the total mass of solid components in the coating solution.

Hydrophilicity was assessed using a contact angle goniometer (OCA 50, DataPhysics, Germany): 10 μL ultrapure water droplets were deposited at room temperature, imaged after stabilization, and analyzed via DSA 1.8 software to determine water contact angles (WCA). For cardiovascular stent applications, PSMASH-coated 316L SS stents (Ø2.85 mm × 18 mm) underwent balloon expansion testing by crimping onto angioplasty balloons, expanding to Ø3 mm at 15 atm for 1 min, followed by SEM examination to evaluate coating adhesion and mechanical integrity.

**Coating adhesion test**

Coat the hydrogel coating evenly on the 316L stainless steel substrate, fix the specimen in the upper and lower clamps of the tensile machine, make sure that the surface of the specimen is perpendicular to the direction of the pulling force, start the tensile machine, apply the pulling force uniformly at a rate of 5 mm/min, record the pulling force-displacement curves, and when the coating is separated from the substrate, record the maximum value of the pulling force.

$\sigma=\frac{Fmax}{A}$(Equation S2)

where A is the cross sectional area of the drawing disc (A=π×(5 mm)^2^).F_max_ stands for maximum tension value.

**Rheological experiments**

The dynamic rheological tests of different hydrogels were characterized at 25°C using a rheometer equipped with 20 mm parallel plates. The hydrogels (diameter: 20 mm, thickness: 2 mm) were loaded into a 0.5 mm gap between the plates and allowed to relax until the normal force was zero. Strain amplitude sweeps (0.01-100%) were first performed to determine the linear viscoelasticity region. Dynamic frequency sweeps were performed at angular velocities ranging from 0.1 to 10 Hz at 1.0% strain amplitude (linear region).

**Characterizing the stability of hydrogel coatings using flow chamber simulations**

To simulate coating stability under hemodynamic conditions, an in vitro degradation assay was performed using a tubular flow chamber chip. The chip's inlet connected to a peristaltic pump via silicone tubing, with the outlet linked to a waste collection tube, forming a closed circulatory loop. Carbon dots (100 μg/mL) were premixed into the hydrogel precursor solution, becoming embedded during free radical polymerization to confer green-light-excitable fluorescence. Coated stainless steel sheets were mounted in the chamber to ensure unimpeded laminar flow. PBS was circulated at 52.21 ml/min (10× physiological blood velocity) using the peristaltic pump^1^. Colloidal adhesion was monitored via laser confocal microscopy at days 0, 3, 7, and 12 by scanning fluorescent signals from the coated surfaces.

**In Vitro degradability of hydrogel**

For the in vitro degradation test, hydrogel bulks, with the same volume (1 mL), were

immersed in 10 mL simulated body fluid (SBF, pH = 7.4-7.5) at constant temperature (37℃) with shaking at 100 rpm to mimic the physiological conditions. At the predetermined time point, hydrogel samples were taken out and rinsed with RO water to remove excess salinity, and they were then lyophilized and weighed. The initial weight of the hydrogel sample (W0) was measured. The hydrogel was then soaked in SBF. At different time points, the samples were retrieved, lyophilized, and weighed (Wt). The degradation rate was calculated using the following formula:

$\text{Degradation ratio = }\frac{\text{W}_{\text{t}}\text{-}\text{W}_{\text{0}}}{\text{W}_{\text{0}}}\text{×100\% }$(Equation S3)

**Puerarin release from hydrogels**

As the hydrogel degrades, the main traditional Chinese herb component puerarin in Pueraria starch is uniformly released, this mechanism effectively addresses the issue of drug burst release and ensures a complete release rate of 100%. The release medium is simulated body fluid, and the samples are placed in a constant temperature oscillating shaker at 37°C. At specific time points, 3 mL samples are taken to measure the absorbance at 250 nm, and the release medium with the same volume, temperature, and pH is replenished.

#### **The in vitro anticoagulant activity and hemocompatibility of hydrogel coatings**

New Zealand rabbits were utilized in this study, with all animal experimental procedures approved by the Animal Ethics Committee of Southwest Jiaotong University and conducted in accordance with the National Institutes of Health (NIH) Guide for the Care and Use of Laboratory Animals. Platelet-poor plasma (PPP) was obtained by centrifuging rabbit venous blood collected in sodium citrate tubes at 3,000 rpm for 15 minutes. Platelet-rich plasma (PRP) was prepared similarly by centrifugation at 1,500 rpm for 15 minutes.

Prothrombin time (PT) and activated partial thromboplastin time (APTT), standard assays for evaluating the extrinsic and intrinsic coagulation pathways respectively, were performed. A 100 μL aliquot of PPP was incubated on each sample surface at 37°C for 30 minutes. The plasma was then aspirated, transferred to a 2 mL Eppendorf tube, mixed with 100 μL of PT reagent, and the coagulation time was recorded using a fully automated coagulation analyzer (Sysmex CS-5100). For APTT measurement, the aspirated plasma was incubated with 100 μL of APTT reagent at 37°C for 5 minutes, followed by the addition of 100 μL of 0.025 mol/L CaCl₂ solution; the time to insoluble fibrin formation was recorded.

Plasma coagulation kinetics were assessed to determine coagulation function within the dynamic range, using 0.025 mol/L CaCl₂ and 125 U/mL heparin (Hep) as positive and negative controls, respectively. A mixture of 40 μL of either PSMA (5%), PSSH (5%), or a 1:1 (v/v) mixture of PSMA and PSSH solution at equivalent concentrations, combined with 60 μL PPP, was added to a 96-well plate. Dynamic measurements were conducted at 405 nm absorbance using a microplate reader (37°C, 60 minutes, 1-minute intervals). The plasma coagulation rate was determined by calculating the slope within the linear range and the maximum slope.

The hemolysis ratio, a critical indicator of blood compatibility for implant materials, was measured. Positive and negative controls employed 0.1% Triton X-100 and 0.9% physiological saline, respectively. Whole blood anticoagulated with sodium citrate was centrifuged twice (2,000 rpm, 5 minutes) and washed with saline. Red blood cells were diluted to 10% with saline and incubated with the test samples (37°C, 60 minutes). The supernatant, obtained after high-speed centrifugation (3,000 rpm, 15 minutes), was analyzed; a 200 μL aliquot was used to measure hemoglobin absorbance at 540 nm. The hemolysis ratio was calculated using the following formula：

$Hemolysis ratio \left( \% \right) =\frac{A_{h-}A_{p}}{A_{t-}A_{p}} \times100\%$ (Equation S4)

where A_h_​, A_p_ and A_t​_represent the absorbance values of the sample supernatant, the negative control (physiological saline), and the positive control (Triton X-100), respectively.

Whole blood clotting time (BCT) and whole blood clotting index (BCI) provide direct assessments of the in vitro anticoagulant properties of the samples. Anticoagulated whole blood was applied dropwise onto the sample surfaces, spread evenly, and incubated at 37°C for 20 minutes. The blood was then aspirated, mixed with CaCl₂ to neutralize the anticoagulant and initiate coagulation, and the clotting time was visually determined by inverting the tube. Alternatively, for BCI determination, anticoagulated whole blood was applied dropwise onto the sample surfaces and spread evenly. Subsequently, 20 μL of CaCl₂ solution was added dropwise to each sample surface, followed by incubation at 37°C for 5 minutes. Then, 2 mL of RO water was added, causing unclotted blood cells on the sample surface to undergo hypotonic lysis. A 200 μL aliquot of the supernatant was analyzed for hemoglobin absorbance at 540 nm. The BCI was calculated using the following formula:

$\mathrm{BCI}\left( \% \right)= \frac{A_{h}}{A_{c}} \times100\%$ (Equation S5)

where A_h_ denotes the absorbance of the sample and A_c_ denotes the absorbance of the control group.

Platelet adhesion and activation were evaluated by incubating the samples with 200 μL of PRP. Following incubation at 37°C for 1 hour, the samples were gently rinsed three times with physiological saline, fixed with 2.5% glutaraldehyde for 30 minutes, and then dehydrated through a graded ethanol series (50%, 75%, 90%, and 100%, 15 minutes per step). After air-drying, the samples were sputter-coated with gold and examined using scanning electron microscopy (SEM) to observe and analyze platelet adhesion morphology and activation state on the surfaces.

**Ex Vivo Circulating Thrombosis Assay**

To evaluate the anticoagulant performance and hemocompatibility of the samples under dynamic blood flow conditions, an ex vivo circulating blood experiment was conducted using New Zealand rabbits. All surgical procedures were performed under aseptic conditions. Catheters were sterilized using ethylene oxide, and samples were sterilized via ultraviolet irradiation. Four adult New Zealand white rabbits (body weight: 2.5–3.0 kg) were utilized in this study. All animals were humanely euthanized using sodium pentobarbital anesthesia.

Initially, rabbits were anesthetized via injection of 3% sodium pentobarbital (1 mL/kg) into the marginal ear vein. Under sterile conditions on a surgical table, the right carotid artery and left jugular vein were dissected and exposed. Blood flow was established through an arteriovenous (AV) extracorporeal circuit (ECC), wherein blood withdrawn from the right carotid artery via an indwelling needle flowed through parallel branches of medical-grade PVC tubing equipped with three-way connectors, before merging and returning to the left jugular vein. Coated samples (10 × 8 × 0.2 mm), pre-curled to fit, were inserted into the PVC tubing. Circulation was maintained for 1 hour without systemic anticoagulation. Following this, the tubing was retrieved and gently flushed with physiological saline. Cross-sectional images of the tubing containing the test samples were acquired. The occlusion rate was calculated by measuring the luminal cross-sectional area before and after circulation. Subsequently, the samples were extracted, weighed, fixed in 2.5% glutaraldehyde, dehydrated, and examined using scanning electron microscopy (SEM) to assess surface morphology and thrombus formation.

**Cell Biocompatibility of Hydrogel Coatings**

Following ultraviolet sterilization, the samples were placed in 24-well plates. Endothelial cells (ECs), RAW 264.7 macrophages, and smooth muscle cells (SMCs) were seeded onto the sample surfaces at a density of 1 × 10⁴ cells per well. On days 1 and 3, the plates were retrieved, and 300 μL of CCK-8 solution (prepared at a 1:9 ratio of CCK-8 reagent to culture medium) was added to each well. The plates were subsequently incubated at 37°C for 1 hour. Following incubation, 150 μL of solution from each well was transferred to a 96-well plate. Absorbance at 450 nm (standard for CCK-8 assays; noted 540 nm in original corrected to standard protocol) was measured using a microplate reader (Varioskan Lux, Thermo Scientific, USA). Cell viability was calculated according to the following formula:

$\mathrm{Cell} \mathrm{viability}= \frac{\mathrm{OD}_{450}\left( E \right)- \mathrm{OD}_{450}\left( B \right)}{\mathrm{OD}_{450}\left( C \right)- \mathrm{OD}_{450}\left( B \right)} \times100\%$ (Equation S6)

Following three washes with physiological saline, Calcein-AM/PI live/dead staining working solution was added to each well. The plates were incubated at 37°C in the dark for 15-30 minutes. Samples were then visualized under a fluorescence microscope: live cells exhibited green fluorescence (Ex/Em = 494 nm / 517 nm), while dead cells displayed red fluorescence (Ex/Em = 535 nm / 617 nm).

For the cell migration assay, SMCs were cultured to the logarithmic growth phase and prepared as a single-cell suspension at a concentration of 1×10⁵ cells/mL. Coated materials (SS, T-SS, PSMA, PSSH, PSMASH) were secured in the lower chambers of Transwell inserts. Subsequently, 300 μL of the cell suspension was added to the upper chamber, while 700 μL of DMEM medium supplemented with 10% FBS was added to the lower chamber. The Transwell system was incubated for 24 hours at 37°C in a 5% CO₂ humidified atmosphere. After incubation, non-migrated cells on the upper membrane surface were gently removed by washing with PBS. Migrated cells on the lower membrane surface were fixed with 4% paraformaldehyde for 15 minutes and stained with 0.1% crystal violet solution for 15 minutes. Excess stain was removed by PBS washing. Migrated cells were imaged using an optical microscope. Five randomly selected fields per sample were photographed, and migrated cells were quantified using Image J software.

**Protective Effect of Hydrogel Coating on Cardiovascular Cells Under Oxidative Stress Model**

The AS microenvironment and vascular stent implantation disrupt vascular homeostasis. Core pathological mechanisms include lipid metabolism disorders, chronic inflammatory responses, endothelial cell dysfunction, and abnormal proliferation/migration of smooth muscle cells (SMCs). During AS progression, macrophage foam cell formation, endothelial cell injury, and SMC phenotypic switching constitute critical events. These aberrant cellular behaviors collectively drive atherosclerotic plaque development and vascular stenosis. Therefore, establishing a robust oxidative stress model to simulate cellular damage and foam cell formation within an atherosclerotic environment enables direct validation of Pueraria lobata polysaccharide hydrogel coating's regulatory effects on cellular lipid metabolism, inflammatory responses, proliferative activity, and phenotypic switching.

Macrophages were seeded in 48-well plates at 5 × 10⁴ cells/mL. After 12 hours, cells were treated with oxidized low-density lipoprotein (ox-LDL, YB-002, Yiyuan Biotechnologies) for 24 hours. ox-LDL concentrations were tested at 0, 20, 40, 50, 60, 80, and 100 μg/mL. A 0.5% (w/v) Oil Red O (Sigma-Aldrich, O0625) stock solution was prepared in isopropanol. The stock solution was mixed with reverse osmosis (RO) water at a 3:2 ratio, allowed to stand, and filtered through a 0.45 μm filter. For staining, cells were incubated with the Oil Red O working solution for 30 minutes, counterstained with hematoxylin for 5 minutes, and destained with isopropanol. Intracellular lipid accumulation was quantified using BODIPY 493/503. Cells were incubated with the working solution at 37°C in the dark for 15 minutes, trypsinized into single-cell suspensions, and analyzed by flow cytometry. For immunofluorescence, fixed cells were permeabilized with 0.1% Triton X-100 for 15 minutes and blocked with 1% bovine serum albumin (BSA) for 2 hours. After washing, cells were incubated with primary antibodies overnight at 4°C, followed by species-specific secondary antibodies for 1 hour at room temperature. Nuclei were counterstained with DAPI for 30 minutes prior to fluorescence imaging.

**Establishment of Atherosclerosis Animal Model**

Control group animals were fed a standard diet, whereas the model group underwent balloon-induced vascular injury followed by continuous feeding with an atherosclerosis-specific high-fat diet (Chengdu Ensiweier Biotechnology Co., Ltd.) for over 14 days. Subsequently, relevant analyses were performed on the animal models: Blood samples were collected from the model animals. Based on preceding experimental results, SS and PSMASH hydrogel-coated stents were selected for implantation studies within the established atherosclerotic animal model. Serum triglycerides (Catalog No. S03027, Raoto) and total cholesterol (Catalog No. S03042, Raoto) levels were quantified using the glycerol phosphate oxidase-peroxidase (GPO-PAP) method. High-density lipoprotein cholesterol (HDL-C, Catalog No. S03025, Raoto) and low-density lipoprotein cholesterol (LDL-C, Catalog No. S03029, Raoto) levels were determined via the direct-selective inhibition method. Glucose concentration (Catalog No. S03039, Raoto) was measured using the glucose oxidase method.

Following euthanasia, the injured vascular segments were fixed in 4% paraformaldehyde. One segment was cryosectioned for Oil Red O staining to visualize lipid deposition, while another segment was embedded, sectioned, and subjected to hematoxylin and eosin (HE) staining to assess lumen loss and pathological changes.

**Vascular Stent Implantation**

Model animals were anesthetized and administered heparin intravenously. The rabbit carotid artery was surgically exposed. Bare-metal SS stents and hydrogel-coated modified stents were implanted into the atherosclerotic lesion sites within the carotid arteries using a percutaneous transluminal angioplasty (PTA) balloon catheter. Postoperatively, penicillin was administered via intramuscular injection for seven consecutive days. Animals were humanely euthanized at 7 days and 28 days post-implantation. Arterial segments containing the stents were harvested and bisected longitudinally using micro-scissors. One segment was fixed in 4% paraformaldehyde for paraffin embedding and sectioning. The other segment underwent electrolytic decalcification to remove the stent metal, enabling soft tissue sectioning and subsequent immunofluorescence staining.

**Characterization of the stented arteries**

The columnar segments in paraformaldehyde solution were further cut transversely, and then dehydrated by gradient alcohols and desiccation in air. Most of them were cured in methyl methacrylate. Afterward, the solidified resins were sliced by a hard-tissue microtome (BQ1600, Lan Ming Medical Treatment, China). The semi-columnar segments in paraformaldehyde solution were further permeabilized with Triton X-100 solution for 3 h and then blocked with BSA solution for 12 h. Subsequently, they were incubated with mouse anti-rabbit CD31 antibody (IgG, Cat. No.: NBP2-44342, 1/100 dilution in PBS) or mouse anti-porcine CD31 antibody (IgG, Cat. No.: NB100-65336, Novus Biologicals, USA, 1/100 dilution in PBS) for 6 h, and then Alexa Fluor® 488-conjugated goat anti-mouse IgG secondary antibody (Cat. No.: abs20013, Absin Bioscience, 1/100 dilution in PBS) for 6 h. After that, they were simultaneously stained with DAPI for 10 min. Finally, the samples were photographed using confocal microscope (Air+, Nikon, Japan) after being extensively washed with PBS. Z-stack images were acquired using a confocal microscope, and CD31 (green), DAPI (blue channel), and brightfield/phase contrast images were acquired and aligned for data analysis using the JACoP plug-in. The remaining semi-columnar segments were dehydrated by gradient alcohols, dried in air, sputter-coated with gold, and then examined by SEM.

H&E staining：After dehydration of paraffin sections, hematoxylin staining for 3-5 min, hydrochloric acid ethanol differentiation, ammonia return to blue, eosin staining for 3-5 min, then gradient ethanol and xylene dehydration and transparency, and finally seal the sections with neutral gum, microscopic observation.

Oil Red O: After a brief rinse in pure water for 5 seconds, the blood vessels were sequentially immersed in 60% isopropanol for 3 seconds and then stained with Oil Red O solution at 37°C in the dark for 60 minutes. Following staining, the vessels were differentiated in 60% isopropanol until they turned pale pink or colorless, and then rinsed under running tap water to stop the differentiation process. Images were captured using an optical microscope.

Immunofluorescence: After dehydration, tissue sections were blocked with 5% goat serum, followed by incubation with the primary antibody at 4°C overnight. After washing, the sections were incubated with a secondary antibody for 1 hour. Nuclei were counterstained with DAPI, and the sections were finally mounted with an anti-fade mounting medium.

Quantitative analysis was performed using ImageJ software. Neointimal thickness and area were measured from H&E images, the lipid-positive area was quantified by color thresholding of Oil Red O stains, and the expression levels of specific markers were assessed by measuring the mean fluorescence intensity in multiple fields of view.

**Statistical Analysis**

All experiments were performed at least three times (n ≥ 3) for each sample. Data normality was assessed by Shapiro-Wilk test (α = 0.05), and homogeneity of variance by Levene's test. Normally distributed data were analyzed by unpaired two-tailed Student's t-test (two groups) or one-way ANOVA with Tukey's post-hoc (multiple groups); non-normal data used Mann-Whitney U test (two groups) or Kruskal-Wallis with Dunn's test (multiple groups). Statistical significance was set at *p* < 0.05. Analyses were performed using GraphPad Prism 9.0.

**
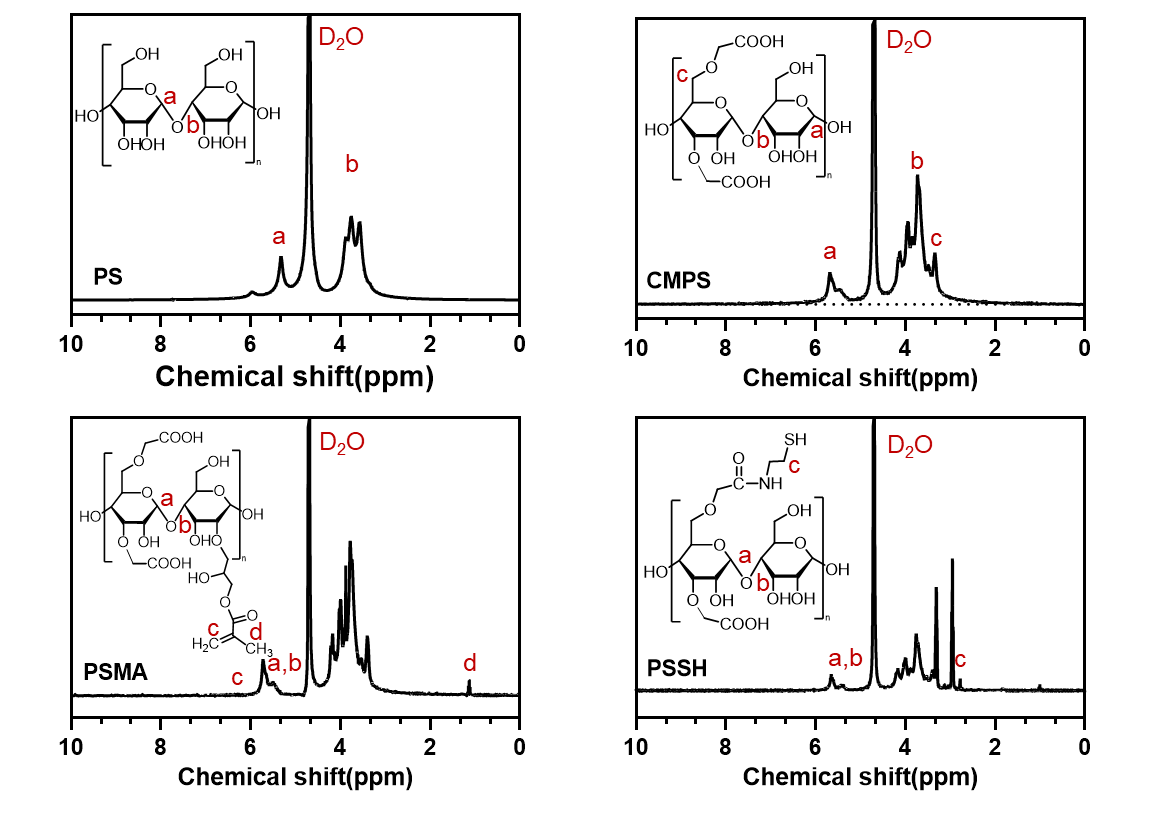

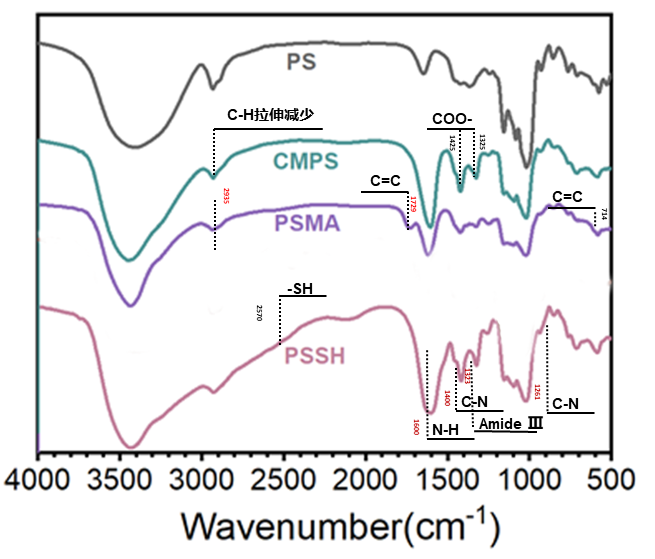
Results and discussion**

Figure S2 H NMR spectra of carboxymethylated, allylated, and thiolated Pueraria

Figure S1 FTIR spectra of carboxymethylated, allylated, and thiolated Pueraria lobata polysaccharide derivatives.

.


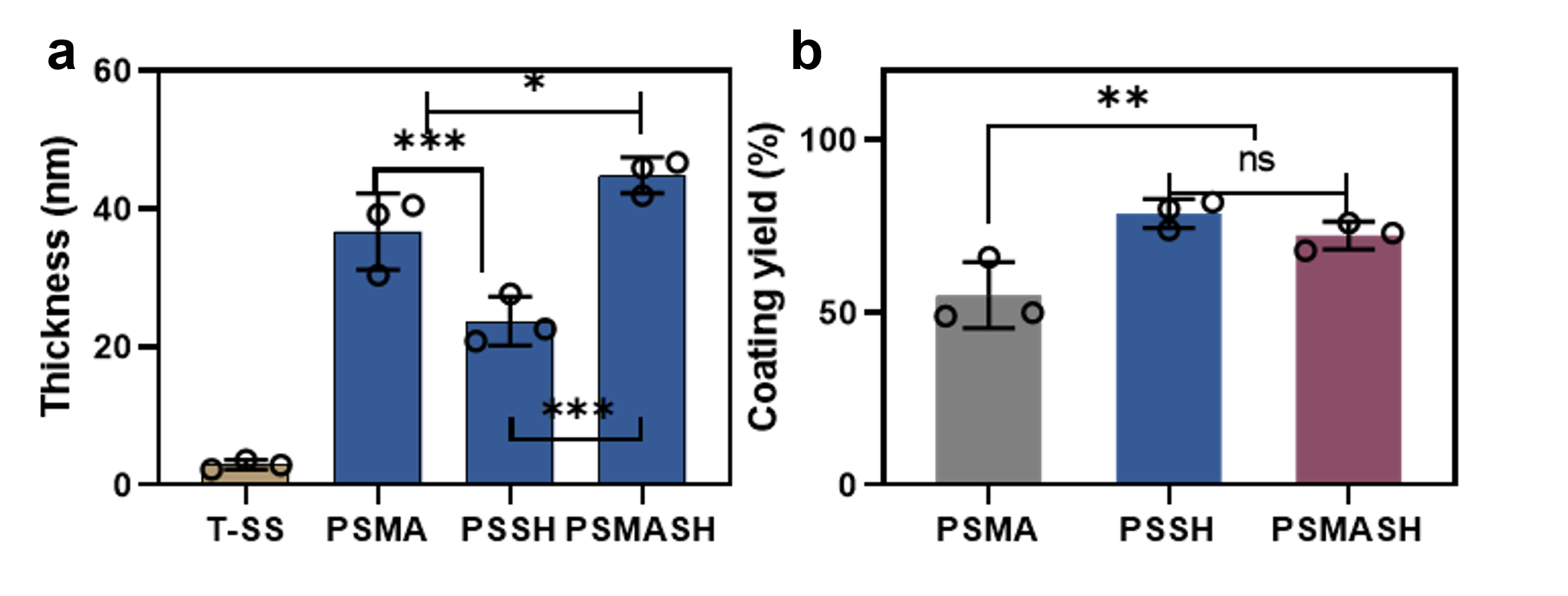
**
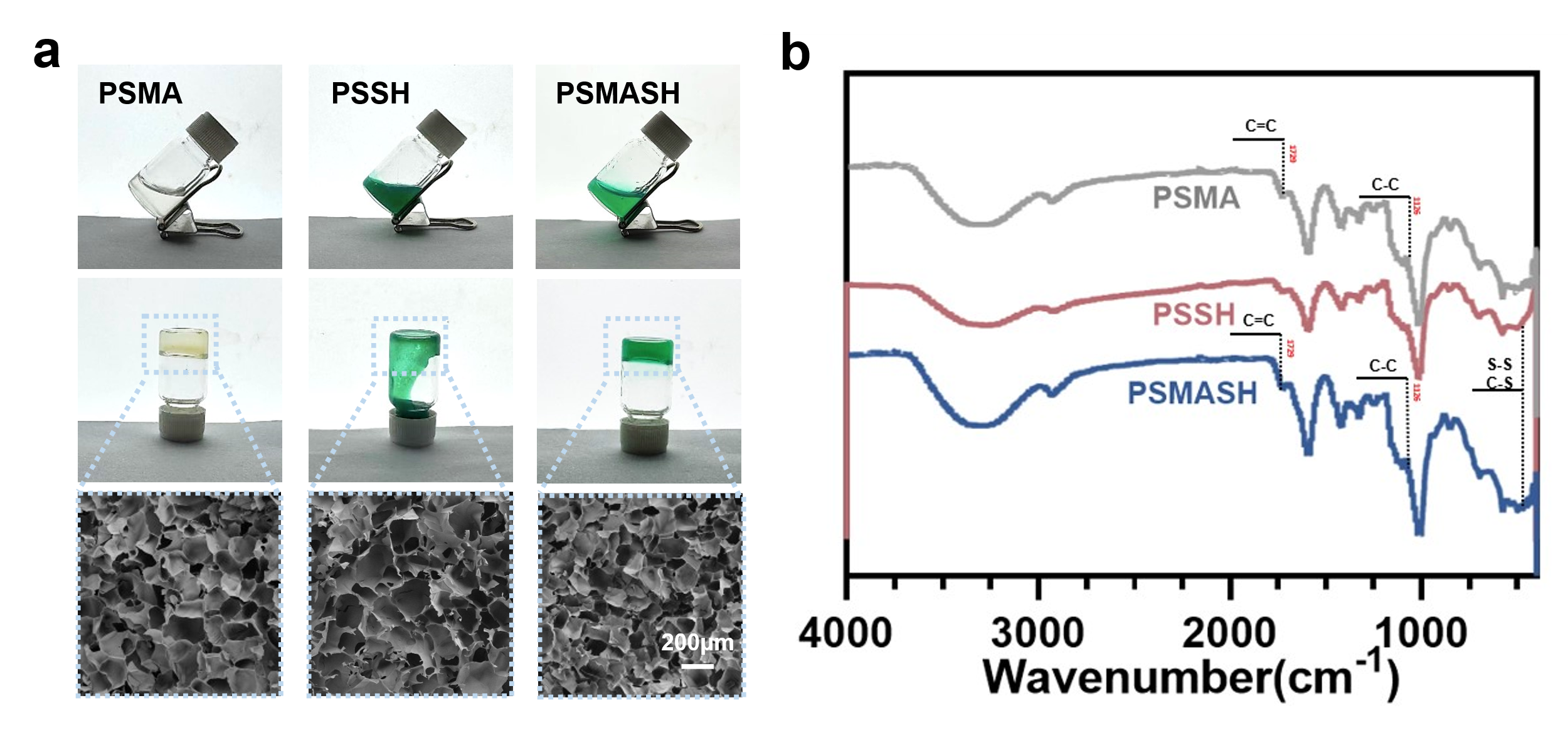

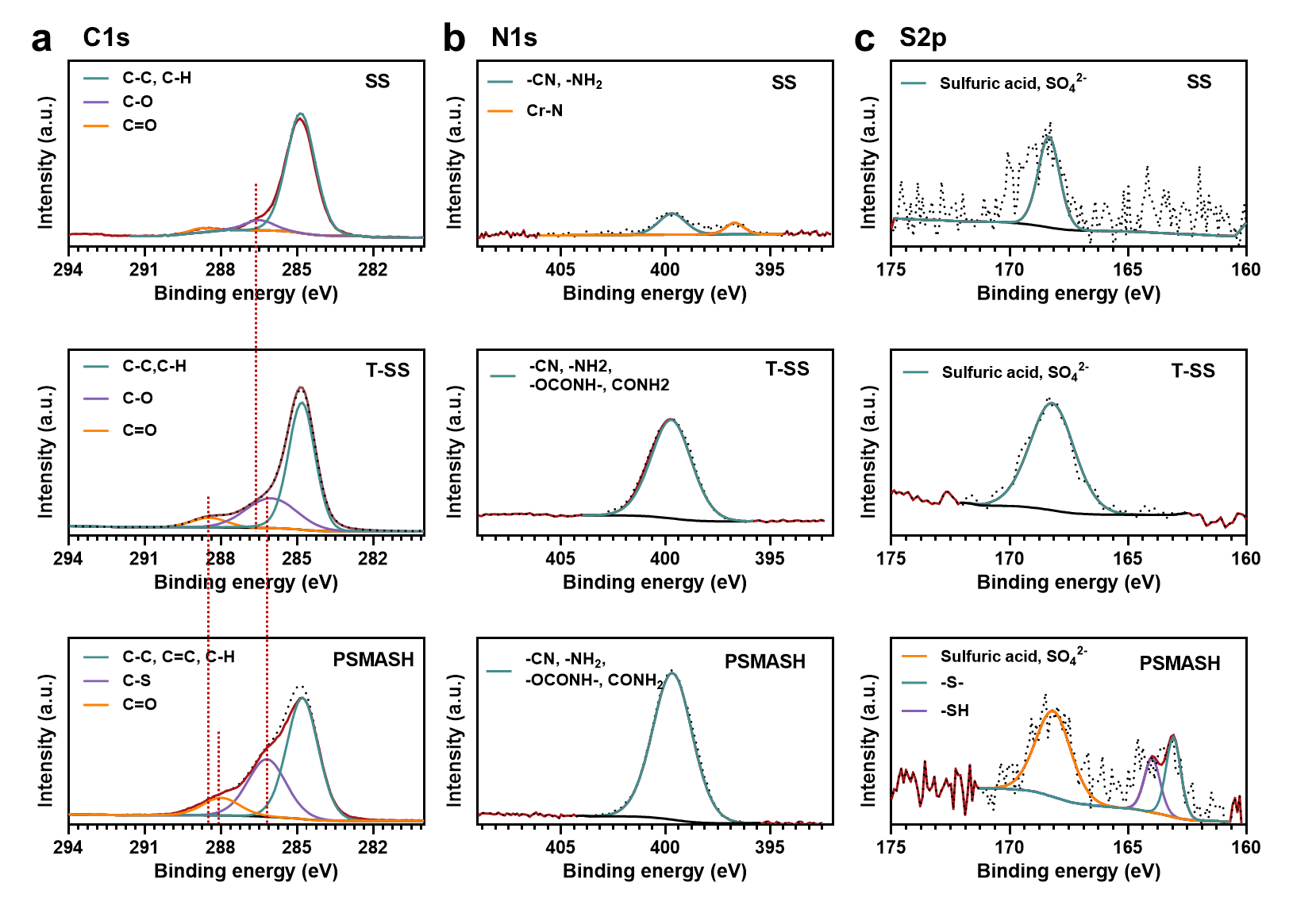
**

Figure S4 (a) Measurement of coating film thickness by spectroscopic ellipsometry; (b) Coating yield

Figure S3 (a) Gelation schematic and cross-sectional SEM images of PSMA, PSSH, and PSMASH hydrogels; (b) FTIR spectra of hydrogel coating surfaces.

Figure S5 High-resolution XPS spectra of SS, T-SS, and PSMASH hydrogel coatings:

(a) C 1s, (b) N 1s, (c) S 2p core-level spectra.

**
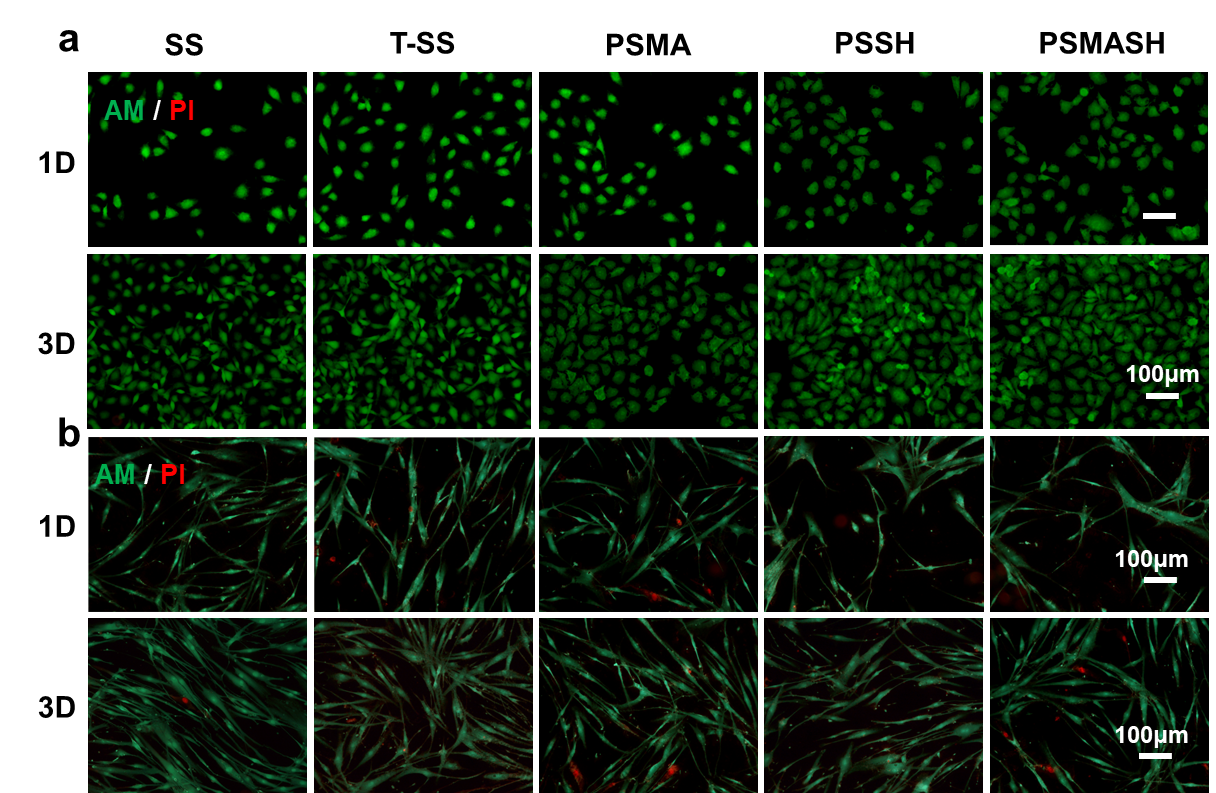

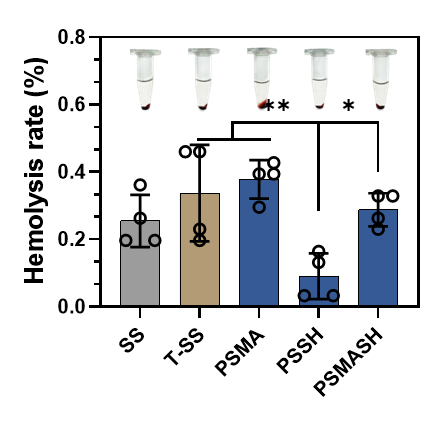

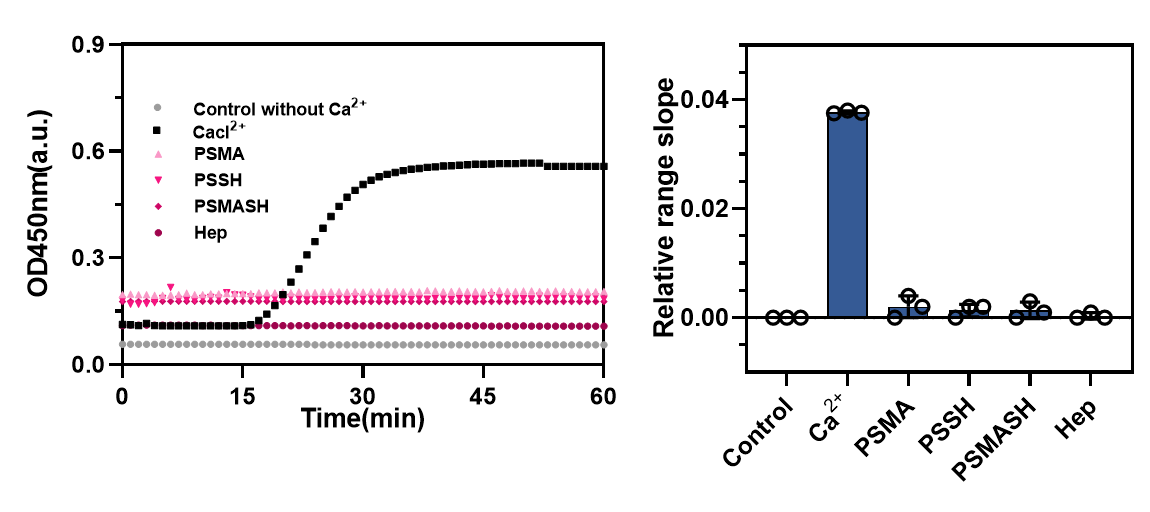
**

Figure S8 Adhesion and proliferation behavior of ECs and SMCs on different coatings. (a) Live/dead staining of ECs on coating surfaces at Day 1 and 3; (b) Live/dead staining of SMCs on coating surfaces at Day 1 and 3(green: live cells; red: dead cells).

Figure S7 Hemolysis ratio of coatings

Figure S6 Plasma coagulation kinetics and maximum slope of coatings in vitro.

**
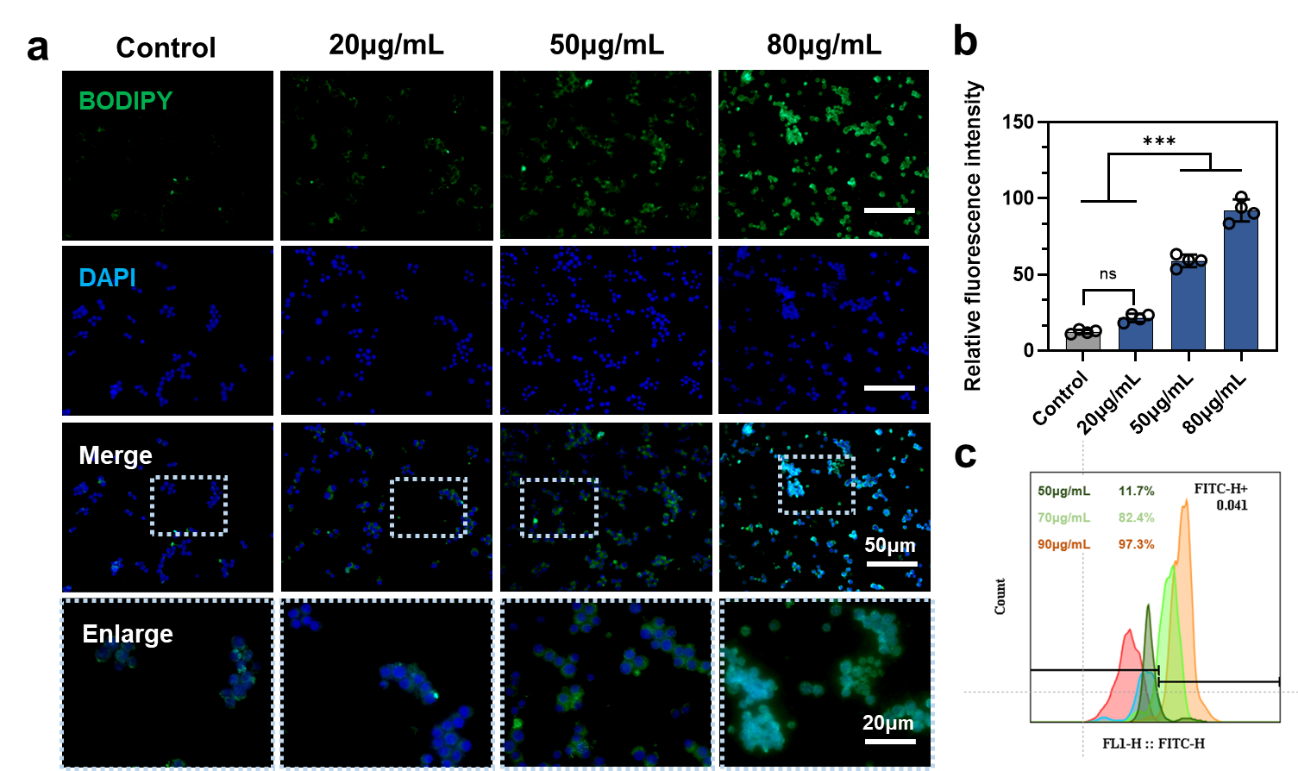

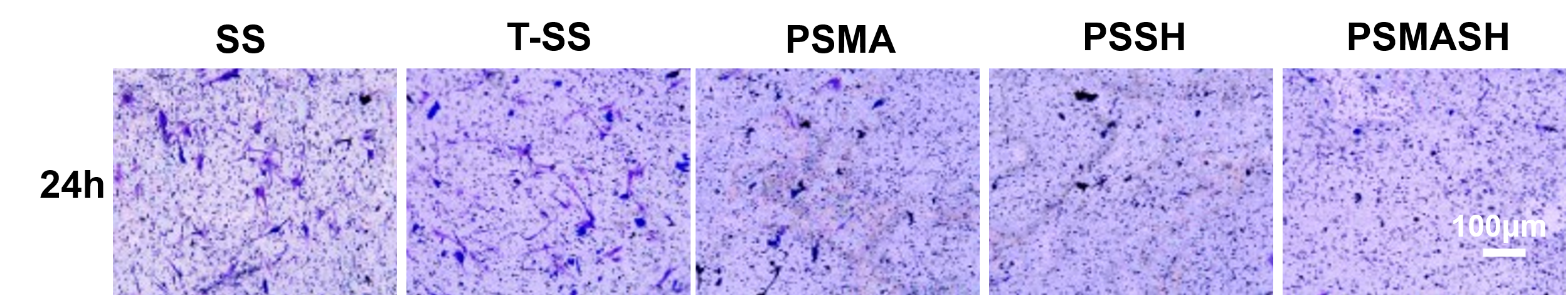

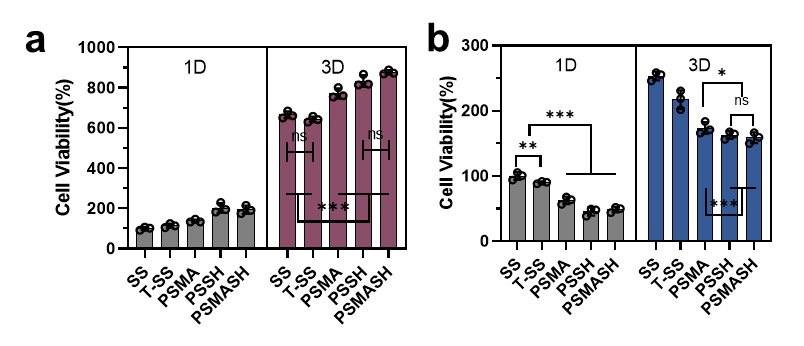
**

Figure S11 Semi-quantitative analysis of RAW 264.7 macrophage foam cell formation and intracellular lipid content under different ox-LDL concentrations. (a) Intracellular lipid staining by BODIPY 493/503 (green); (b) Semi-quantitative fluorescence intensity of BODIPY; (c) Flow cytometry quantification (n = 4; ***p < 0.001).

Figure S9 Cell viability of ECs and SMCs on various coatings. (a) Viability of ECs on coating surfaces at Day 1 and 3; (b) Viability of SMCs on coating surfaces at Day 1 and 3.

Figure S10 SMC Migration Images


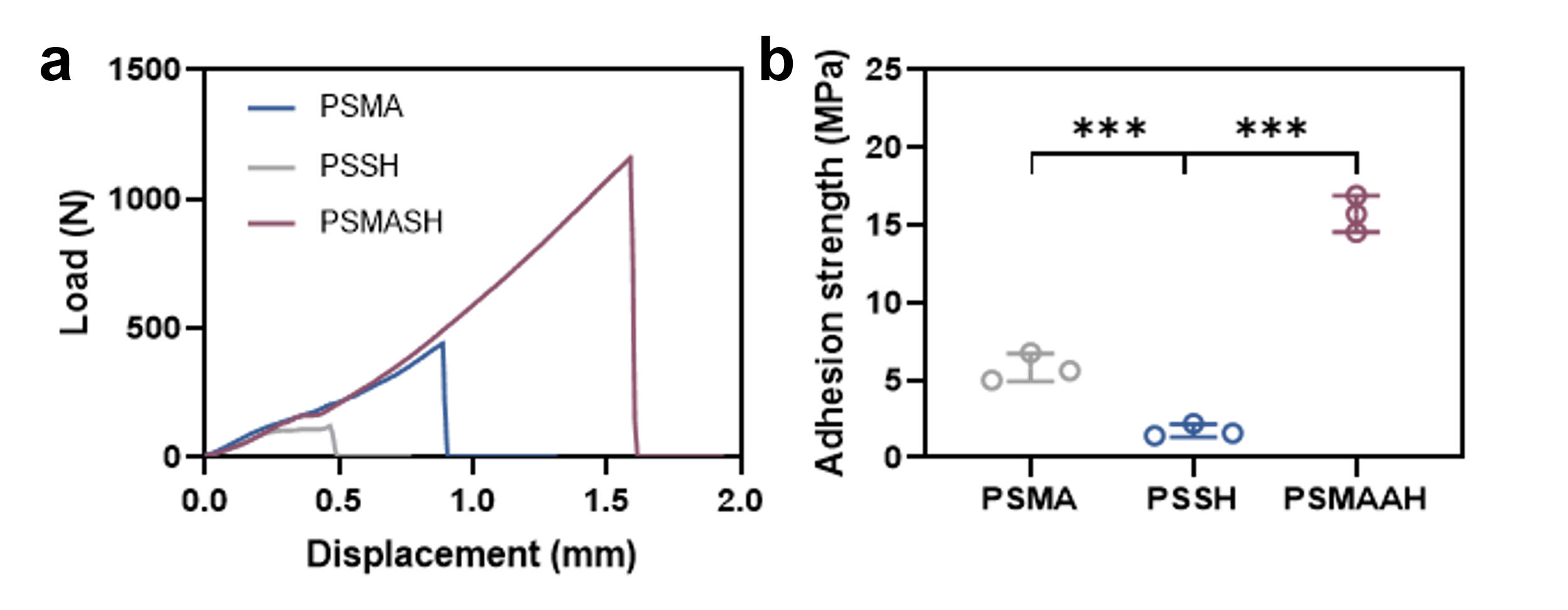

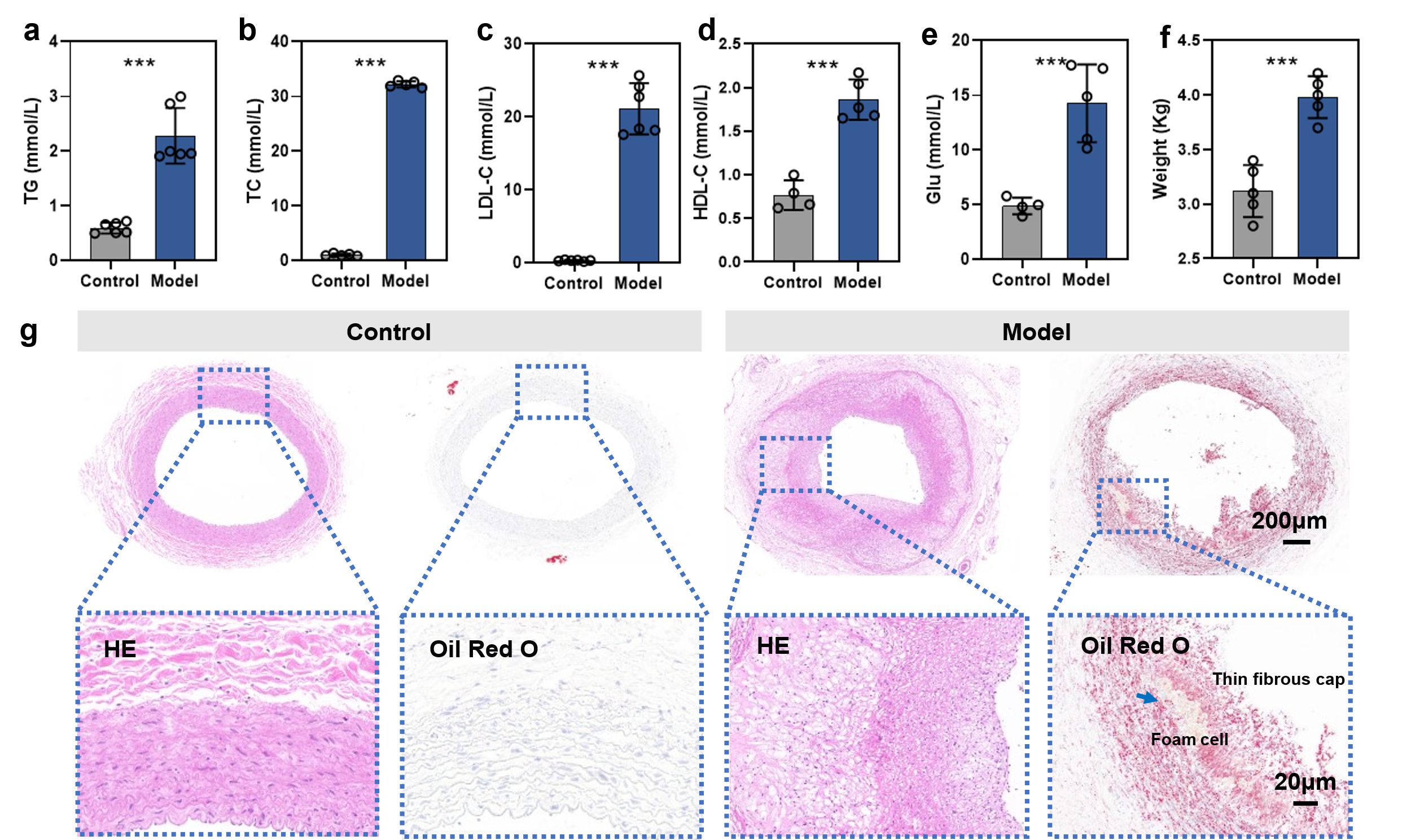


Figure S13 Adhesion of hydrogel coatings. (a) adhesion; (b) adhesion strength

Figure S12 Blood parameter alterations in animal models. (a) Triglycerides (TG); (b) Total cholesterol (TC); (c) Low-density lipoprotein cholesterol (LDL-C); (d) High-density lipoprotein cholesterol (HDL-C);

(e) Blood glucose concentration (Glu); (f) Body weight changes; (g) Representative biochemical sections and pathological staining of atherosclerotic lesions.


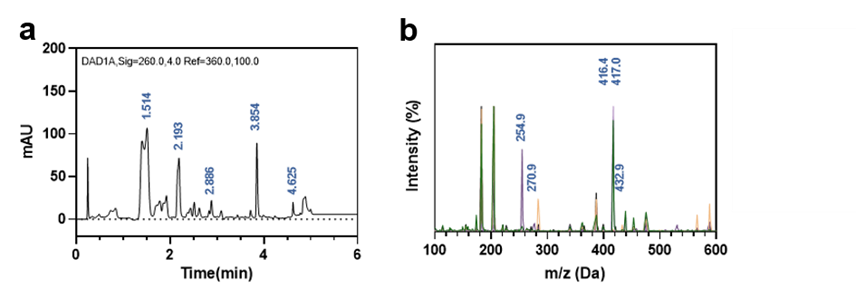

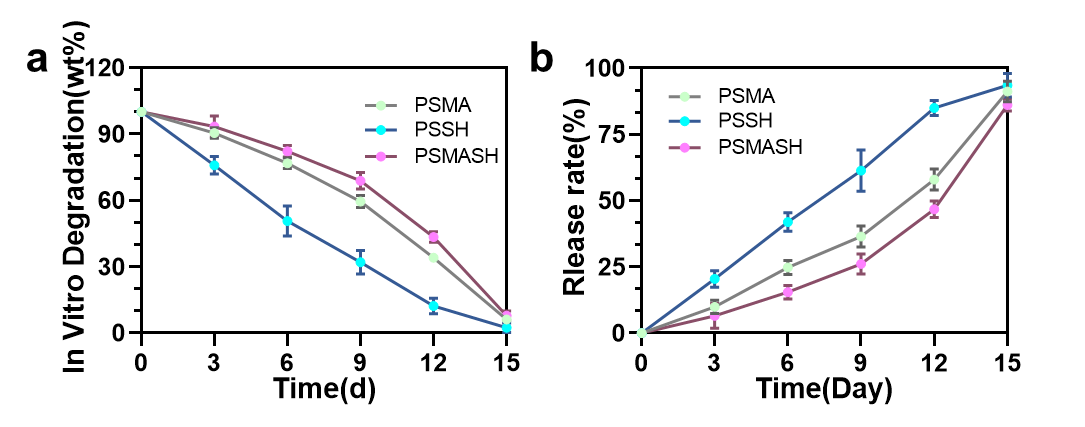

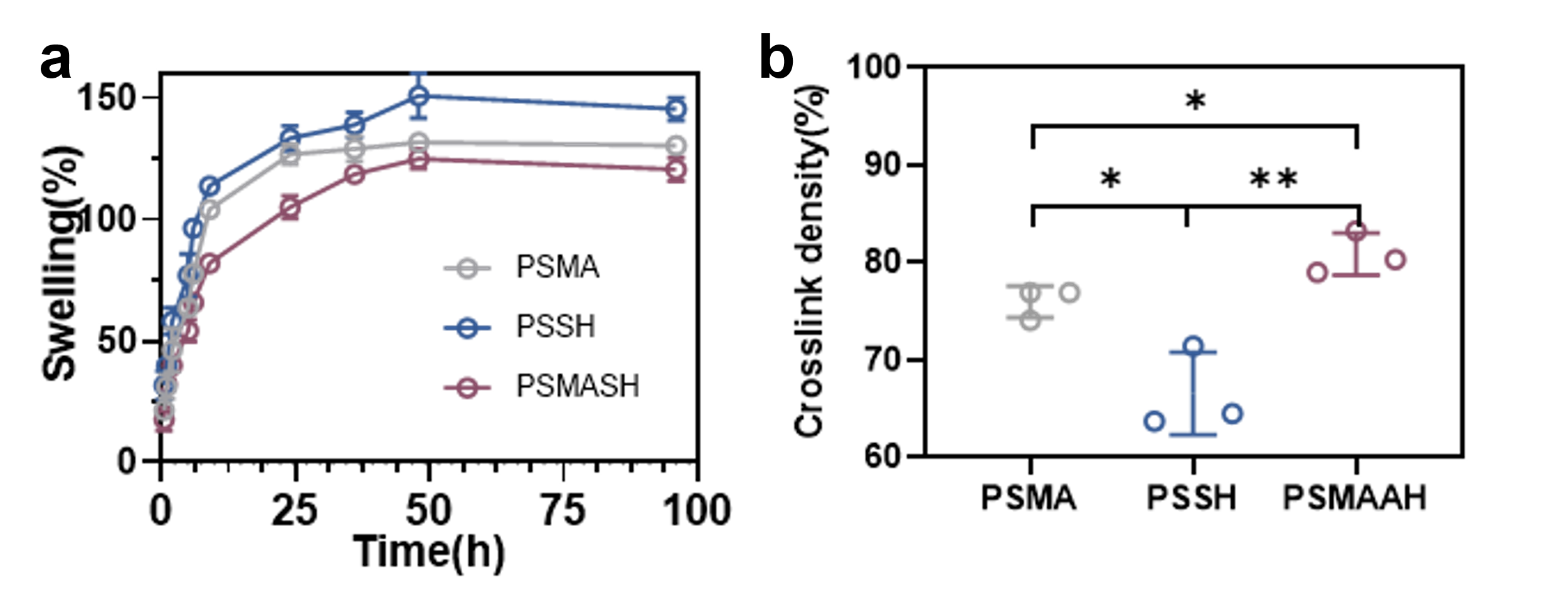


Figure S17 HPLC profiles of Pueraria lobata polysaccharides after ethanol reflux extraction. (a) HPLC plot of the sample; (b) mass spectrometry results lobata polysaccharide derivatives

Figure S16 Degradation of PSMA, PSSH, PSMASH hydrogels and release rate of puerarin in simulated body fluid

Figure S15 Rheological properties of hydrogels; storage modulus (G′), loss modulus (G″)

Figure S14 (a) hydrogel 96h swelling results; (b) crosslink density

1. Shionoya, S., Noninvasive diagnostic techniques in vascular disease. *Int Angiol* **1987,** *6* (3), 213-221.
